# How Epitranscriptomic Machinery Senses Environmental Cues

**DOI:** 10.1002/adbi.70154

**Published:** 2026-08-31

**Authors:** Shayan Jalali, Ehsan Pashay Ahi

**Affiliations:** ^1^ Department of Health Sciences (DISS) University of Eastern Piedmont/Piemonte Orientale (UPO) Novara Italy; ^2^ Faculty of Biological and Environmental Sciences University of Helsinki Helsinki Finland; ^3^ Faculty of Medicine University of Turku Turku Finland

**Keywords:** environmental cues, epitranscriptomics, metabolic coupling, post‐translational modifications, RNA modifications, signal‐dependent regulation

## Abstract

Environmental fluctuations remodel RNA modification landscapes, yet the routes that connect cue detection to writer–eraser–reader control remain dispersed across disciplines. Here, we consolidate upstream mechanisms capable of driving epitranscriptomic change and organize them by response speed. At the fastest proximal level, catalytic output can be modulated through shifts in substrate and cofactor availability, redox and ionic state, temperature, and direct chemical or metal interference with enzyme active sites, although transcriptome‐wide RNA readouts may appear later. Over minutes to hours, cue‐responsive signaling can reach the machinery through post‐translational modification, partner switching, subcellular trafficking, and stress‐induced condensates that may gate access to modified transcripts. Across hours to days, regulator abundance and specificity are reshaped by transcriptional programs, translational control, and protein quality‐control pathways, enabling adaptation and, in some contexts, persistence. We propose a kinetics‐to‐sensors approach for interpreting time‐resolved epitranscriptomic datasets and prioritizing perturbations that discriminate among candidate upstream inputs. We also outline conceptual gaps and experimental practices needed to establish causal cue‐to‐mark chains.

## Graphical abstract

Exogenous exposures, microenvironmental signals, and intracellular metabolic or redox states can alter RNA‐modification machinery through catalytic chemistry, signaling‐dependent PTMs, compartmental redistribution, or changes in abundance and composition. Here, environmental “sensing” means cue‐dependent changes in writer, eraser, reader, or accessory‐factor behavior, not necessarily direct receptor‐like cue detection. Machinery responses and detectable RNA‐modification changes may occur on different timescales because RNA turnover, compartmentalization, cell‐state heterogeneity, and assay limits can delay or obscure RNA readouts. Stoichiometry‐aware, RNA‐age‐aware, compartment‐resolved, machinery‐state, and single‐cell measurements can help link RNA changes to proximal machinery events. Arrows indicate conceptual information flow, not a single linear pathway. One cue may engage multiple mechanisms, and timing helps prioritize mechanisms but does not establish causality. Abbreviations: 2OG, 2‐oxoglutarate; PTMs, post‐translational modifications; R‐2HG, R‐2‐hydroxyglutarate; ROS, reactive oxygen species; SAH, S‐adenosylhomocysteine; SAM, S‐adenosylmethionine.

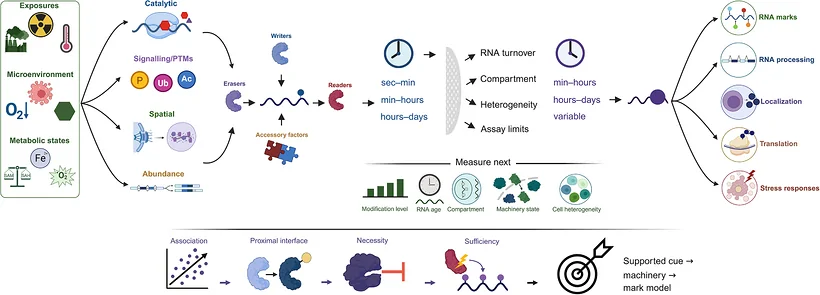

## 1 Introduction

More than 170 chemically distinct ribonucleoside modifications have been catalogued across RNA classes, and together they define the epitranscriptome as a regulatory layer that complements transcriptional and chromatin‐based control [1]. RNA modification profiles have been linked to fundamental cell functions and to disease phenotypes, and clinical relevance has been underscored by the deliberate use of modified nucleotides in therapeutic RNAs [2, 3, 4, 5, 6]. Rapid methodological progress (including improved sequencing‐based mapping, quantitative approaches, and emerging engineering strategies) has moved the field from descriptive maps toward causal questions about how marks are installed, removed, and interpreted in context [7, 8].

The field often describes epitranscriptomic regulation through modular “writer–eraser–reader” systems, in which enzymes deposit or remove marks and dedicated binding proteins translate them into changes in RNA processing, stability, localization, and translation [9, 10, 11, 12]. N6‐methyladenosine (m6A) has been treated as a paradigmatic example because of its prevalence and the breadth of its described functions, while multiple non‐m6A marks in mRNA (including Ψ, m6Am, m1A, inosine, m5C, ac4C, Nm and internal m7G) have been increasingly emphasized for their distinct regulatory logics and often low, context‐dependent stoichiometries in mRNA [10, 13]. RNA modifications also operate within broader physiological programs, such as metabolic remodeling and immune adaptation, where RNA fate control is intertwined with cellular state [14, 15, 16, 17]. RNA modification systems are well positioned to participate in environmental responses because they regulate RNA fate after transcription. This allows cells to adjust translation, RNA stability, localization, splicing, and ribonucleoprotein assembly using transcripts that are already present or newly synthesized during the first wave of stress adaptation. Such regulation can be transcript‐selective, compartment‐specific, and compatible with both rapid responses and longer‐lived adaptation. In this sense, the epitranscriptome provides a flexible layer between environmental sensing and gene‐expression output, complementing transcriptional and protein‐level control rather than replacing them.

A growing body of work has connected environmental variation to altered RNA modification landscapes, with evidence spanning chemical exposures, physical stressors, and pathophysiological microenvironments [18, 19, 20, 21]. In exposure‐focused syntheses, changes have been described not only in modification abundance but also in the expression of writer/eraser/reader proteins, raising the possibility that epitranscriptomic machinery functions as an early molecular mediator of environmental inputs, downstream of primary cue detection but upstream of RNA fate regulation [18, 19]. Toxicology‐centered reviews have further argued that environmentally linked disorders may be shaped by m6A‐dependent regulatory tiers, while also noting inconsistencies that complicate mechanistic inference from endpoint measurements [22]. More recent surveys of environmental toxins (e.g., heavy metals, air pollutants, pesticides, and endocrine‐disrupting chemicals) have reinforced that multiple marks and multiple regulatory tiers can be perturbed, but have also highlighted that causal coupling routes remain incompletely resolved [23].

Selected systems demonstrate environmental responsiveness even on short timescales, supporting the premise that upstream coupling can occur far faster than de novo transcriptional reprogramming. Heat shock has been associated with rapid enrichment of m6A within 5′ UTRs of newly transcribed stress‐induced transcripts and with selective translation outcomes, implying that stress‐driven nuclear relocalization of the reader YTHDF2 and reduced access of the eraser FTO can reshape marking patterns [24]. Ultraviolet irradiation triggers m6A accumulation at DNA damage sites within minutes, suggesting that acute physical stimuli can drive localized marking events through fast regulation of writer and eraser activities [25]. By contrast, sustained hypoxia illustrates a slower coupling route: HIF‐dependent induction of the m6A demethylase ALKBH5 requires transcriptional regulation and has been linked to altered methylation of defined transcripts [26]. Thus, environmental responsiveness can be rapid, delayed, or mixed, depending on whether the cue acts through pre‐existing machinery, signaling‐dependent rewiring, or new regulator production.

Environmental stress can also reorganize the RNA–protein landscape and thereby modulate how marks are interpreted. m^6^A‐containing transcripts enhance phase separation of m6A‐modified RNAs with YTHDF proteins, supporting a mechanism by which stress‐induced condensates could gate access to RNAs and modify functional readouts without necessarily requiring large global shifts in mark abundance [27]. In parallel, redox imbalance and oxidative stress have been reviewed as contexts in which RNA methylation programs are reshaped during cellular adaptation and therapy resistance, reinforcing that “environment” can be operationalized as intracellular chemistry as well as external exposure [28]. In immunity‐related environments, A‐to‐I editing by ADAR1 acts as a safeguard that tunes dsRNA‐sensing pathways, underscoring that nucleic‐acid‐associated immune cues, including viral RNA and endogenous dsRNA‐like species such as repeat‐derived or mislocalized self RNAs, can be buffered by RNA‐modifying enzymes that function at the interface of self–nonself discrimination [29]. In plants, RNA modifications have been described as contributors to growth and stress responses, indicating that cue‐responsive epitranscriptomic regulation is not restricted to animal systems [30].

**FIGURE 1:**
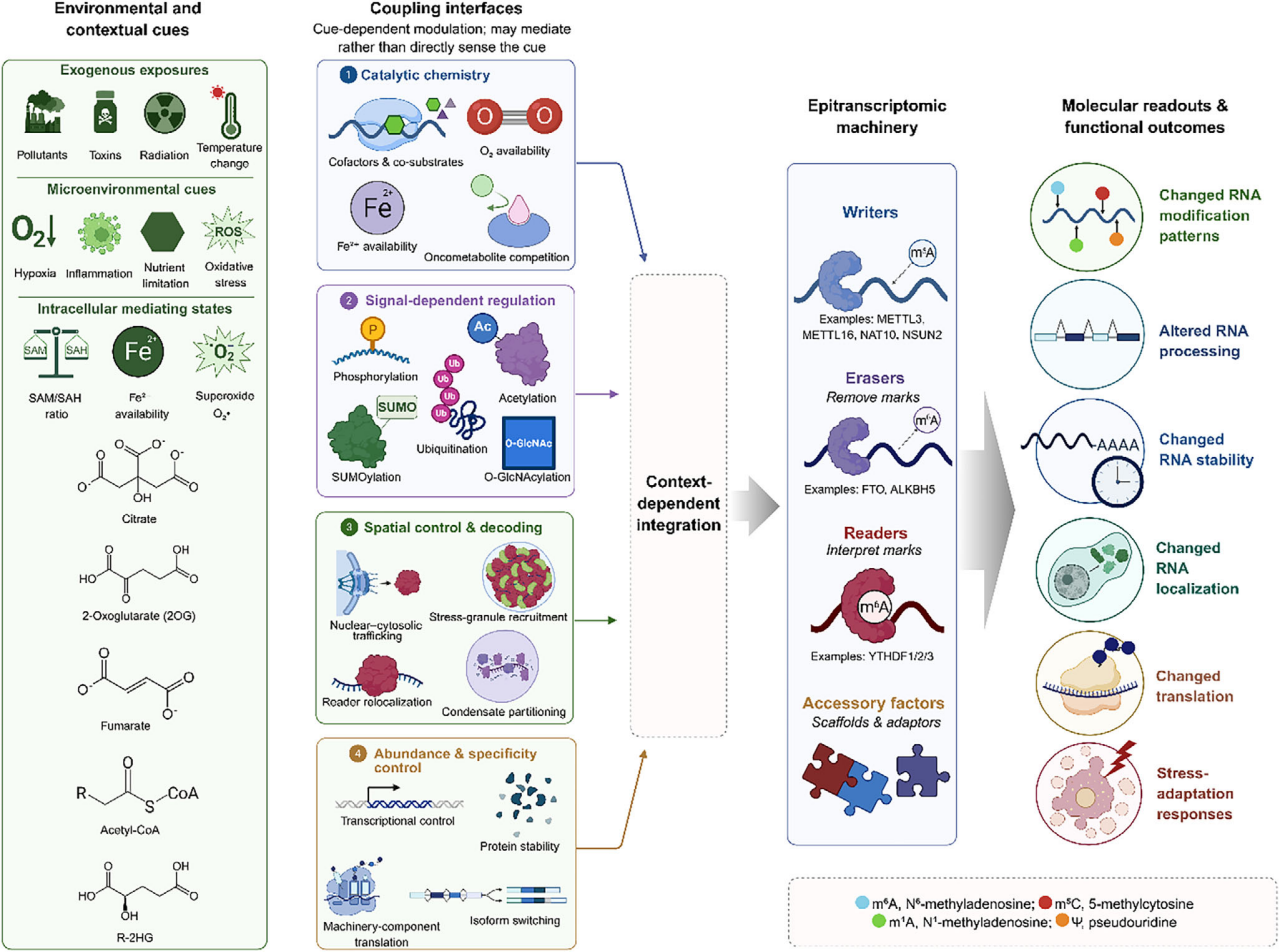
Environmental cue‐to‐machinery coupling in epitranscriptomic regulation. Environmental and contextual cues, including exogenous exposures, tissue or microenvironmental conditions, intracellular mediating states, and nucleic‐acid signals such as viral RNA or endogenous double‐stranded RNA, may influence epitranscriptomic machinery through four broad interfaces: catalytic chemistry, signal‐dependent regulation, spatial control and decoding, and abundance or specificity control. Here, “sensing” means cue‐dependent alteration of machinery behavior, not necessarily direct receptor‐like cue detection. These interfaces are overlapping and non‐exclusive; arrow colors indicate interface modules rather than fixed machinery targets. Cue integration may depend on cell type and state, cue dose and duration, metabolic context, subcellular compartment, RNA substrate or age, and machinery abundance or state. Writers install RNA modifications, erasers remove selected modifications, readers/decoders interpret selected modified RNAs, and accessory factors regulate localization, assembly, recruitment, stability, substrate specificity, or catalytic activity. Not all RNA modifications have established erasers or dedicated readers. The pathways shown are representative, not exhaustive, and evidence strength varies across mechanisms, marks, and systems. Inclusion of metabolites and machinery components does not imply that every cue‐to‐machinery relationship is established in vivo, and biochemical dependence alone does not demonstrate acute cellular regulation. RNA‐level regulation may provide rapid, reversible, transcript‐selective control linking environmental cues to RNA consequences and cellular responses. SAM/SAH, S‐adenosylmethionine/S‐adenosylhomocysteine balance; 2OG, 2‐oxoglutarate; R‐2HG, R‐2‐hydroxyglutarate; ROS, reactive oxygen species; m^6^A, N^6^‐methyladenosine; m^5^C, 5‐methylcytosine; m^1^A, N^1^‐methyladenosine; Ψ, pseudouridine.

Despite this expanding evidence base, mechanistic synthesis has tended to be partitioned by mark type, disease area, or exposure category, and the specific question of how environmental cues are sensed and coupled into writer/eraser/reader behavior has remained scattered [7, 18, 19, 22, 23]. Two upstream‐control themes have been repeatedly emphasized but seldom integrated with exposure biology: first, metabolite and cofactor dependence, through which fluctuations in nutrient state and metabolic flux can alter reaction energetics and enzymatic output [31, 32]; and second, post‐translational regulation of RNA‐modifying proteins, through which signaling inputs can reshape activity, stability, localization, and complex assembly [33, 34]. A response‐kinetics‐based approach is useful because the timescale on which a modification shift appears can constrain the set of plausible upstream coupling routes that fit the data [24, 25, 26, 27, 28, 31, 32, 33, 34]. Accordingly, this Review organizes upstream mechanisms that connect environmental cues to epitranscriptomic machinery as a kinetics‐aware continuum. We define environmental cue sensing as a change in a proximal property of writers, erasers, readers, or their accessory factors, rather than as direct ligand detection by the enzymes themselves. We separate exogenous exposures, physiological microenvironmental conditions, and intracellular chemical states, and ask how each can alter catalytic activity, localization, complex assembly, reader engagement, or regulator abundance (Figure 1). Finally, we use response speed to prioritize candidate mechanisms and discriminating perturbations for mechanistic testing.

## 2 Scope and Terminology: What It Means for Epitranscriptomic Machinery to Sense Environmental Cues

In this Review, environmental cues are defined broadly as physical, chemical, and physiological perturbations that alter cellular chemistry or signaling state, including but not limited to toxicant exposure, nutrient availability, temperature shifts, radiation, oxidative stress, inflammatory milieus, and oxygen limitation [2, 19, 23]. To avoid conflating distinct sources of regulation, we distinguish exogenous environmental exposures, physiological or tissue microenvironmental conditions, and intracellular biochemical states that transmit or reflect these inputs. Exogenous exposures include toxicants, pollutants, metals, radiation, and temperature shifts. Physiological or tissue microenvironmental conditions include hypoxia, inflammatory cytokines, nutrient limitation, oxidant burden, and local metabolic stress. Intracellular biochemical states, such as SAM/SAH balance, acetyl‐CoA availability, 2OG‐linked metabolite ratios, labile iron, or redox state, are included only when they function as proximal coupling interfaces between a cue and the RNA‐modifying machinery. Thus, baseline gene activity, mutations, or metabolic fluctuations are not treated as environmental cues by default. A second distinction concerns exposure duration. Acute laboratory perturbations, such as heat shock, irradiation, nutrient withdrawal, or short‐term oxidative stress, are especially useful for identifying early biochemical, signaling, or localization‐dependent events. By contrast, chronic toxicant exposure, sustained hypoxia, inflammatory milieus, and prolonged nutrient limitation often combine early machinery regulation with later transcriptional, translational, and proteostatic remodeling. These longer contexts are therefore treated as mixed‐kinetic settings unless the timing of the upstream machinery change has been resolved experimentally.

Here, epitranscriptomic machinery encompasses proteins and complexes that (i) install chemical marks (writers), (ii) remove or chemically reverse marks where reversibility has been demonstrated (erasers), and (iii) decode marks through selective binding or altered ribonucleoprotein assembly (readers), together with accessory factors required for localization, substrate selection, or catalytic output [2, 7, 13, 35]. The scope is not limited to m6A regulators; rather, machinery associated with non‐m6A mRNA marks (Ψ, m6Am, m1A, inosine, m5C, ac4C, Nm, internal m7G) is also considered, because regulation, stoichiometry, and dynamics have been emphasized as defining features for these marks and their effectors [13, 18]. The operational inclusion of editing (e.g., A‐to‐I) is justified when cue‐linked modulation has been supported by evidence at the level of the modifying enzyme and its substrate recognition, even if writer/eraser language is not traditionally used for that pathway [2, 18]. Because m^6^A is the most prevalent internal modification in eukaryotic mRNA and has the most developed writer–eraser–reader literature, many mechanistic examples in this Review are drawn from m^6^A studies. This emphasis reflects the current state of the field rather than an assumption that m^6^A principles apply uniformly to all RNA modifications. The broader model proposed here remains mark‐inclusive, but its application to Ψ, ac^4^C, m^5^C, inosine, Nm, m^1^A, m^6^Am, internal m^7^G, and other marks must account for mark‐specific chemistry, stoichiometry, reversibility, RNA‐class distribution, and assay maturity.

Sensing is used as a shorthand for cue‐to‐machinery coupling and is not intended to imply that RNA‐modifying enzymes necessarily function as primary receptors for environmental ligands [2, 7, 35]. Instead, we infer sensing when a defined cue reproducibly alters at least one proximal property of the machinery, such as activity, substrate access, complex assembly, localization, or abundance, or when it measurably alters mark deposition/removal in a manner consistent with such proximal changes [2, 7, 31, 35]. This framing is aligned with recent syntheses in which dynamic changes in RNA modifications and in their regulatory proteins have been discussed as environmentally responsive layers whose mechanistic routes remain incompletely resolved [19, 23].

Several boundaries are imposed to keep the discussion mechanistically focused. First, downstream consequences of RNA marks (e.g., altered translation programs, RNA decay, or phenotype‐level stress tolerance) are treated as outcomes rather than as “sensing” itself unless direct coupling to machinery regulation has been established [2, 18]. Second, cue‐linked correlations in steady‐state mark maps are not treated as mechanistic evidence unless confounding by transcript abundance, RNA turnover, and cell‐state composition has been explicitly addressed [7, 35]. Third, the term “direct” is reserved for cases in which a cue is shown to affect catalytic chemistry or substrate/cofactor availability without requiring intermediate signaling, transcriptional, or translational changes, whereas “indirect” is reserved for pathways in which signaling or transcriptional programs are required to reconfigure the machinery [31, 32, 33].

**FIGURE 2:**
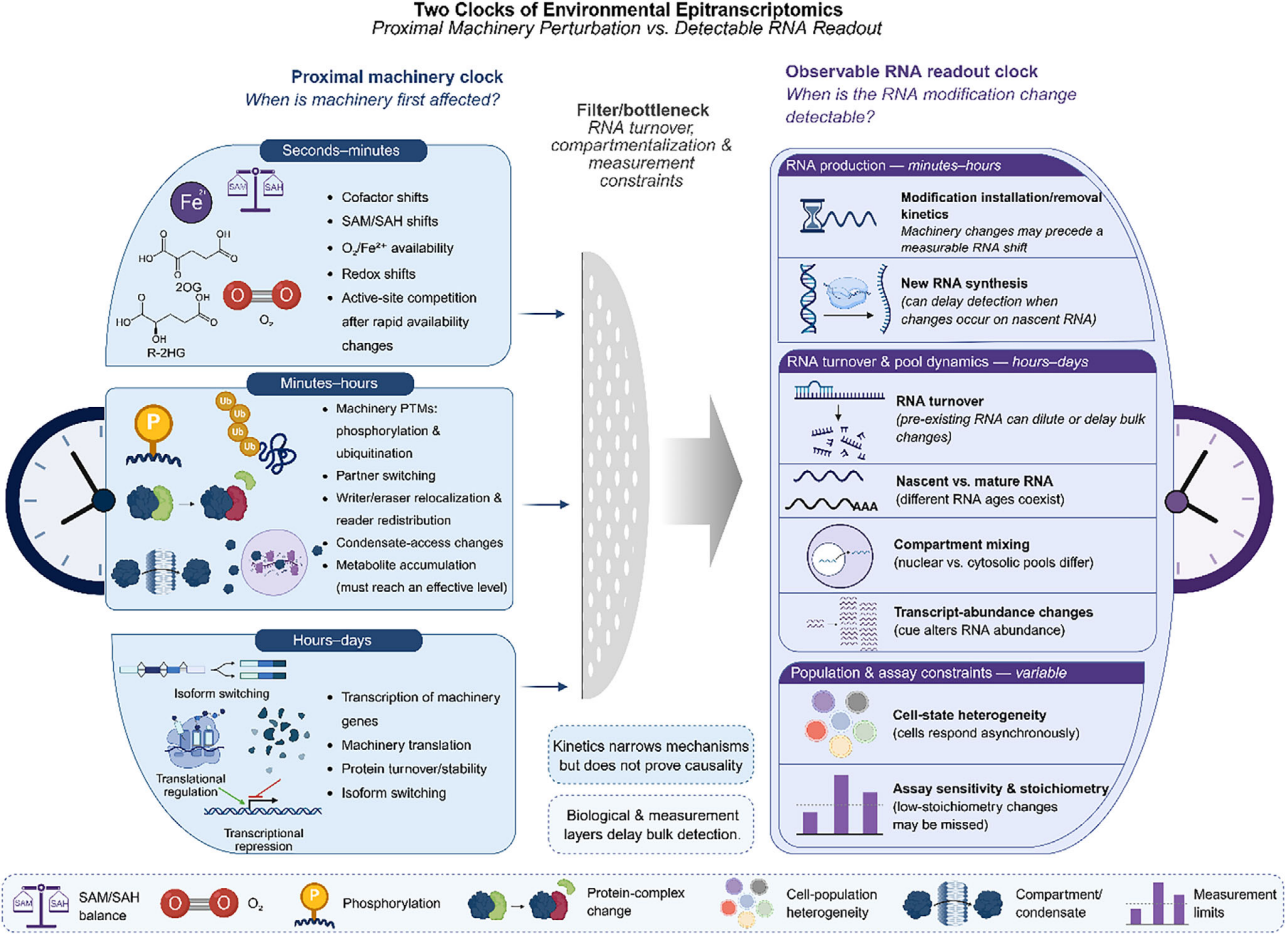
Two clocks of environmental epitranscriptomics: proximal machinery perturbation versus detectable RNA readout. The proximal machinery clock indicates when an environmental cue can first alter epitranscriptomic machinery activity, localization, interactions, or abundance; the observable RNA readout clock indicates when the resulting RNA‐modification change becomes experimentally detectable. These clocks can diverge. Rapid proximal effects may reflect changes in cofactors, cosubstrates, oxygen, iron, redox state, or active‐site competition once the relevant metabolite reaches an effective concentration, whereas cue‐driven production or accumulation of metabolites such as R‐2HG may require minutes to hours. Intermediate responses may involve PTMs, partner switching, relocalization, reader redistribution, or condensate‐associated access, whereas longer responses may involve transcription, translation, protein turnover, or isoform switching. Detectable RNA‐modification changes may lag because of installation/removal kinetics, new RNA synthesis, dilution by pre‐existing RNA, RNA turnover, nascent and mature RNA pools, compartment mixing, transcript‐abundance changes, cell‐state heterogeneity, or assay sensitivity. Thus, machinery perturbation can precede a measurable bulk RNA shift. Apparent response onset is assay‐dependent, and key modification changes should be confirmed using an orthogonal measurement principle. The indicated time windows are approximate, overlapping, and context‐dependent; they prioritize candidate mechanisms rather than define rigid boundaries or establish causality. PTM, post‐translational modification; SAM/SAH, S‐adenosylmethionine/S‐adenosylhomocysteine balance; 2OG, 2‐oxoglutarate; R‐2HG, R‐2‐hydroxyglutarate. Stoichiometry refers to the fraction of RNA molecules carrying a modification at a defined site or within a defined RNA population.

A kinetics‐based vocabulary is adopted because response speed can constrain plausible coupling routes [36]. Fast coupling is defined as changes that can occur within seconds to minutes after cue application, a timescale that is most consistent with direct biochemical modulation (e.g., altered substrate/cofactor pools, redox state, ionic strength, or direct chemical interference with active sites) or, in some cases, with rapid post‐translational regulation and relocalization of pre‐existing machinery [2, 31, 32, 33, 37]. Intermediate coupling is defined as changes emerging over minutes to hours, a window in which signaling‐dependent post‐translational modification, partner switching, and stress‐induced condensates are typically invoked as gating mechanisms for access to marked transcripts or for reader deployment [31, 38]. Slow coupling is defined as changes appearing over hours to days, a window more compatible with transcriptional and translational regulation of machinery abundance, proteostatic remodeling, and longer‐lived adaptive states [2, 19, 23]. This distinction between the proximal machinery clock and the observable RNA readout clock is central to the approach used here (Figure 2).

These kinetic categories, summarized with upstream coupling interfaces and representative examples in Tables 1 and 2, describe the earliest plausible point at which the machinery can be affected, not necessarily the time at which a transcriptome‐wide modification change becomes detectable. A direct biochemical input, such as metabolite competition at an active site, may be proximal and rapid once the metabolite reaches the relevant enzyme pool, but its RNA‐level readout can lag if the metabolite accumulates gradually, compartmental access is limited, or modified RNA populations turn over slowly. Conversely, an apparently slow bulk response can reflect asynchronous fast responses in a subset of cells. This distinction between the proximal machinery clock and the observable RNA readout clock is central to the framework used here. Interpretation of response speed is complicated by the fact that epitranscriptomic measurements are usually performed on RNA populations whose composition changes over time. Modification dynamics can reflect (i) altered methylation or demethylation rates on existing transcripts, (ii) selective marking of newly synthesized RNA, or (iii) altered decay of modified versus unmodified RNA species, any of which can shift apparent modification levels without requiring rapid enzymatic turnover on mature transcripts [37, 38, 39]. Time‐resolved mapping on nascent RNA shows that m6A can be deposited co‐transcriptionally and transiently, indicating that early windows can capture deposition events that steady‐state measurements may miss [39]. Complementary isotope‐ and mass‐spectrometry‐based strategies quantify turnover of modified ribonucleosides and help separate transcriptional from post‐transcriptional contributions to apparent mark changes under nonstationary conditions [37]. For these reasons, kinetics is treated here as a constraint on hypotheses rather than as a stand‐alone diagnostic of mechanism [7, 35, 37].

**TABLE 1:** Kinetics‐tiered upstream coupling interfaces for epitranscriptomic machinery. Conceptual taxonomy linking observed response speed to the most plausible proximal coupling interfaces that regulate writer/eraser/reader activity, access, localization, or abundance. Use this table to shortlist mechanisms before deep mechanistic dissection.

Kinetic regime (typical window)	Proximal coupling interface	What changes first (proximal readout)	What to measure early	Discriminating perturbations	Key refs.
**Fast** (seconds–minutes)	**Methyl donor/product balance** (SAM/SAH)	Writer catalytic feasibility/substrate engagement	Metabolites (SAM/SAH), writer occupancy/activity	Methionine/SAM depletion or rescue; MAT2A hairpin perturbation	[31, 32, 40, 41, 42]
**Fast** (seconds–minutes)	**Acetyl donor availability** (acetyl‐CoA)	Acetyltransferase reaction potential	Acetyl‐CoA pools; ac4C (MS)	Carbon flux modulation; NAT10 perturbation	[43]
**Fast** (seconds–minutes)	**Fe(II)/2OG/O_2_ dependence**	Dioxygenase eraser/oxidase catalytic flux	O_2_ tension, labile iron, 2OG‐related metabolites; mark turnover	Hypoxia/reoxygenation; iron chelation/rescue; 2OG analogs	[44, 45, 46, 47]
**Fast‐proximal; delayed cellular readout possible**	**Competitive metabolite inhibition** (e.g., R‐2HG, fumarate, citrate)	Direct active‐site competition once metabolite is present	Oncometabolites; rapid enzyme activity proxies	Metabolite add‐back/removal; enzyme mutants; competitive inhibitors	[46, 47, 48, 49]
**Fast** (minutes)	**RNA structure/access changes under physical cues**	Enzyme–RNA engagement and target accessibility	RNA structure proxies; site‐specific induction	Temperature shifts; enzyme targeting mutants	[50, 51, 52]
**Intermediate** (minutes–hours)	**Kinase‐driven phosphorylation**	Machinery activation + relocalization	PTM state; subcellular fractionation; local mark deposition	ATM/ATR/MAPK inhibitors; phosphosite mutants	[25, 53, 54, 55]
**Intermediate** (minutes–hours)	**SUMOylation/ubiquitin circuitry**	Reader/eraser affinity, activity, and/or stability	SUMO/Ub status; binding assays; decay rates	SUMO pathway perturbation; deubiquitinase inhibition; lysine mutants	[33, 34, 55, 56, 57]
**Intermediate** (minutes–hours)	**Acetylation/O‐GlcNAcylation**	Localization routing; stability	PTM state; nuclear/cytosolic distribution	HDAC/HAT or OGT/OGA perturbation; PTM‐site mutants	[58, 59, 60]
**Intermediate** (minutes–hours)	**Condensate‐gated decoding/access**	Reader redistribution; RNA partitioning	Stress granule markers; reader relocalization; partitioning	Condensate disruption; reader mutants; translation inhibition controls	[38, 61, 62, 63]
**Slow** (hours–days)	**Cue‐activated transcriptional programs**	Regulator abundance (writers/erasers/readers)	Proteomics; mRNA/protein time courses	Block transcription/translation; TF pathway inhibition	[26, 64, 65]
**Slow** (hours–days)	**Proteostasis/E3 ligase control**	Machinery turnover (dose control)	Ubiquitination; half‐life assays; proteasome dependence	Proteasome inhibition; E3 ligase perturbation; stability mutants	[66, 67, 68]
**Slow** (hours)	**Isoform switching/compartment rewiring**	Isoform abundance + interactome shifts	Isoform‐resolved proteomics/RNA; localization	IFN pathway perturbation; isoform‐specific depletion	[69]

**TABLE 2:** Cue‐to‐machinery‐to‐mark exemplars that anchor each kinetic regime. Representative, well‐specified cue‐to‐machinery chains spanning fast, intermediate, and slow regimes. Use these as anchor cases to interpret time‐resolved datasets and to select discriminating perturbations that test proximal coupling.

Cue/context	Machinery interface	Machinery change shown	Mark/output phenotype	Timescale class	Key refs.
Methionine starvation / SAM depletion	SAM‐sensing by METTL16 via MAT2A hairpins	Altered METTL16 engagement/output	METTL16‐dependent control of MAT2A processing and SAM homeostasis	Fast→ Intermediate	[40, 41, 42]
R‐2HG accumulation (IDH‐mutant contexts)	Competitive inhibition of FTO	Reduced demethylation capacity	Increased m6A with downstream expression programs	Direct biochemical interface; cellular readout may be delayed	[48]
FH loss → fumarate accumulation	Competitive metabolite pressure on 2OG enzymes	Inhibition‐consistent remodeling without major expression shifts	Increased global mRNA m6A; selective enzyme sensitivity	Direct biochemical interface; delayed cellular/readout kinetics	[49]
Citrate (structural inhibition)	Active‐site competition at 2OG pocket	Direct inhibition shown structurally/biochemically	Reduced ALKBH5 demethylase activity	Fast biochemical inhibition shown in vitro; cellular timing context‐dependent	[46, 47]
Heat shock (yeast)	RNA structure/access‐dependent targeting	Inducible Pus7p targeting	Rapid stress‐linked pseudouridylation program	Fast	[50, 51, 52]
DNA double‐strand breaks	ATM‐dependent phosphorylation	METTL3 activation/relocalization to damage sites	Local m6A and YTHDC1 recruitment supporting HR	Intermediate	[53]
Irradiation/DNA damage	ATR‐dependent phosphorylation	YTHDF1 nuclear retention via export control	Splicing regulation of DDR transcripts; radioresistance	Intermediate	[54]
MAPK/ERK signaling environments	Phosphorylation + USP5 deubiquitination	Writer complex stabilization	Increased m6A; fate control in model systems	Intermediate	[55]
Oxidative stress (ROS)	Phosphorylation → SUMOylation cascade	ALKBH5 activity inhibited via substrate access effects	Increased m6A with DDR program effects	Intermediate	[56]
Hypoxia	SUMOylation of reader	Increased YTHDF2 binding/decay capacity	Enhanced decay of m6A‐modified mRNAs	Intermediate	[57]
IL‐6 / inflammatory milieu	Acetylation‐dependent routing	METTL3 deacetylation → nuclear translocation	Increased nuclear m6A output linked to metastasis programs	Intermediate	[58]
Nutrient/O‐GlcNAc flux	O‐GlcNAcylation‐dependent routing	YTHDF1 localization altered via export machinery	Target expression shifts tied to localization	Intermediate	[59]
Sustained hypoxia	Transcriptional induction	HIF‐dependent ALKBH5 upregulation	Transcript‐selective m6A remodeling (e.g., NANOG)	Slow	[26]
Hyperinflammation	NF‐κB transcriptional control	WTAP upregulation/assembly propensity	Increased global m6A; inflammatory phenotypes	Slow	[64]
Proteostasis stress states	E3 ligase‐mediated turnover	METTL14 degradation tuned by STUB1 (protected by METTL3)	m6A homeostasis via “dose control”	Slow	[66]
Ferroptosis	Proteasomal control of non‐m6A writer	STUB1‐mediated NSUN2 degradation	Reduced m5C on Gpx4 mRNA; ferroptosis promotion	Slow	[67]
IFN signaling	Isoform switching + interactome rewiring	ADAR1p150 induction and interaction changes	Editing capacity positioned at dsRNA‐sensing interface	Slow	[69]

Methodological limitations are also treated as part of the terminology, because “change in modification” can refer to distinct observables depending on the assay used. Many widely used sequencing‐based approaches provide enrichment‐ or signal‐based proxies that are not inherently stoichiometric, which complicates inference about magnitude and timing of mark changes, particularly when transcript abundance shifts in parallel [7, 35, 70]. Recent reviews have therefore highlighted stoichiometry, cellular heterogeneity, and environmental responsiveness as distinct axes of epitranscriptomic description and have emphasized that quantitative and single‐molecule strategies (including nanopore direct RNA sequencing) are reshaping what can be concluded about modification dynamics [7, 35, 71]. Benchmarking initiatives and challenge‐based comparisons now improve comparability and reliability in computational detection of modifications from nanopore signals, further underscoring that “presence” and “fraction modified” are not interchangeable terms across platforms [72].

Condensate‐based regulation is treated as a specific mechanistic category rather than as a generic stress correlate. Stress granules and related biomolecular condensates have been defined as rapidly inducible assemblies formed under diverse stress conditions, and their potential to gate RNA availability and reader engagement has been discussed as a plausible route by which environment can be coupled to interpretation of marks [38, 61]. At the same time, this interpretation requires caution because transcript features such as length and translation status strongly influence stress granule enrichment, and m6A‐related variables explain only limited variance in direct tests; “mark‐dependent partitioning” should therefore be treated as context‐dependent rather than assumed [38, 62]. Condensate‐gated access/decoding is therefore used only when reader redistribution, binding requirements, or functionally relevant partitioning has been supported by direct experimentation [38, 62].

Finally, the phrase “upstream mechanism” is restricted to causal links from cue to machinery state (or to mark installation/removal) and is not extended to every signaling pathway altered by stress. Post‐translational modification of RNA‐modifying proteins is treated as an upstream control layer when cue‐responsive signaling is shown or argued to modulate enzyme stability, localization, or catalytic competence through phosphorylation, ubiquitination, acetylation, SUMOylation, or related modifications [33]. Metabolite and cofactor dependence is treated as an upstream control layer when changes in one‐carbon flux, SAM/SAH balance, α‐ketoglutarate (2‐oxoglutarate) availability, oxygen tension, or metal cofactors are implicated in controlling reaction flux of writers or erasers [31, 32, 37]. Under this terminology, the central aim is not to recapitulate stress signaling broadly, but to catalogue the interfaces through which sensing is plausibly implemented at the machinery level, organized by the timescale on which those interfaces can act.

## 3 Fast Coupling: Direct Biochemical Modulation of Writer/Eraser/Reader Activity

Fast environmental coupling should primarily involve mechanisms that do not require new transcription or translation, because local chemical or physical inputs can alter enzymatic steps as soon as they reach the machinery. In bulk RNA measurements, however, “fast” responses can appear later because RNA turnover, compartmentalization, metabolite accumulation, and assay kinetics delay the detectable readout. This section therefore focuses on coupling routes with rapid proximal control points, even when cellular or transcriptome‐wide effects are detected later.

### 3.1 Cofactor and Cosubstrate Dependence as Built‐in Chemical Interfaces

Most RNA methyltransferases have been constrained by the availability and competitive environment of the methyl donor S‐adenosylmethionine (SAM) and its product inhibitor S‐adenosylhomocysteine (SAH), thereby creating a direct biochemical interface between one‐carbon metabolism and methyl‐mark installation [31, 32]. Mechanistically, SAM supplies the methyl group, whereas SAH is the reaction product that can inhibit methyltransferases. A decrease in SAM, an increase in SAH, or a lower SAM: SAH ratio can therefore reduce methylation potential without requiring a change in writer abundance. METTL16 provides the clearest example of a dedicated SAM‐sensing mode: MAT2A RNA hairpins couple intracellular SAM depletion, for example during methionine starvation, to altered METTL16 occupancy, methylation output, and downstream MAT2A processing, thereby tuning SAM homeostasis through an m6A‐dependent circuit [40, 41, 42]. In this setting, the metabolite pool functions as the cue, and sensing occurs through catalytic competence and RNA engagement rather than through a separate receptor [40, 41, 42].

Other writer systems whose core reactions require small‐molecule donors may use analogous chemical interfaces. For example, NAT10‐catalyzed N4‐acetylcytidine (ac4C) installation on mRNA uses acetyl‐CoA as the acetyl donor, making acetyl‐CoA availability a plausible biochemical variable rather than an established acute regulator of mRNA ac4C dynamics [43]. Although the dominant physiological timescales for transcriptome‐wide ac4C changes have varied across contexts, direct evidence that short‐term acetyl‐CoA fluctuations drive mRNA ac4C remodeling remains limited; therefore, ac4C is discussed here as a candidate metabolic interface that requires further testing [43].

On the eraser side, several RNA demethylation and oxidation reactions rely on Fe(II)/2‐oxoglutarate (2OG)‐dependent dioxygenases, which link catalytic flux to oxygen tension, iron availability, and the balance between 2OG and structurally related tricarboxylic acid (TCA) intermediates. These enzymes use Fe(II), 2OG, and molecular oxygen to oxidize methylated bases or related chemical groups. As a result, low oxygen, altered iron availability, or accumulation of metabolites that compete with 2OG can reduce eraser or oxidase activity even when enzyme expression is unchanged. FTO has been established as an Fe(II)/2OG‐dependent demethylase of N6‐methyladenosine‐related marks (including m6A and cap‐adjacent m6Am) [44, 73], and ALKBH5 has been established as an additional mammalian m6A demethylase in the same enzymatic superfamily [45]. Because these enzymes require oxygen and Fe(II) for catalysis, rapid shifts in oxygen tension or labile iron pools have been expected to alter demethylation capacity without requiring changes in enzyme abundance [44, 45, 46, 47]. Beyond m6A, related chemistry has been used for additional RNA modification pathways, extending the same chemical sensing logic to other marks and RNA classes [74, 75, 76, 77].

### 3.2 Competitive Metabolite Inhibition and Oncometabolite Control of Dioxygenase Erasers

Multiple systems support direct inhibition of RNA‐targeting dioxygenases by endogenous metabolites, in which accumulation of TCA‐derived or TCA‐related metabolites alters RNA methylation without corresponding changes in writer or eraser expression. These examples are classified as direct biochemical interfaces because the metabolites can act at or near the catalytic core once present, but the cellular timescale of the response depends on metabolite accumulation, compartmental access, and RNA turnover. The key biochemical idea is competitive access: 2OG supports dioxygenase catalysis, whereas 2OG‐like or TCA‐related metabolites can occupy the same catalytic environment and slow demethylation. When demethylation slows, methylated RNA can persist or accumulate as RNA molecules are newly synthesized or degraded. R‐2‐hydroxyglutarate (R‐2HG) inhibits FTO activity, increases m6A levels, and produces downstream transcriptomic consequences in sensitive leukemia and glioma contexts, providing a clear example of a metabolite that chemically modulates an epitranscriptomic eraser [48]. More recently, fumarate accumulation driven by fumarate hydratase loss has been shown to increase global mRNA m6A in hereditary leiomyomatosis and renal cell carcinoma models, with minimal evidence for altered abundance of core methyltransferase components or demethylases, supporting an inhibition‐based mechanism consistent with competitive pressure on 2OG‐dependent RNA demethylases [49]. In that study, selective sensitivity across RNA‐modifying 2OG‐dependent enzymes has also been highlighted by the finding that mitochondrial tRNA f5C biology was not detectably perturbed under fumarate accumulation, arguing that metabolite inhibition can be mark‐ and enzyme‐specific rather than uniform across the dioxygenase family [49].

Direct binding of common metabolites to dioxygenase active sites has provided a structural basis for such inhibition. Citrate has been identified as an inhibitor that can occupy the 2OG‐binding pocket of ALKBH5 and disrupt its demethylase activity, as shown by co‐crystal structures and catalytic assays [46]. Related structural work has indicated that citrate can also occupy 2OG‐binding pockets in closely related dioxygenases under relevant buffer conditions, reinforcing that central carbon metabolites can compete at the catalytic core when concentrations and compartmental access are permissive [47]. Together, these observations have supported a model in which environmental or microenvironmental changes that reshape intracellular metabolite ratios can be coupled to RNA demethylation potential through direct active‐site competition once the relevant metabolite is present, with downstream mark changes emerging as RNA populations are renewed [46, 47, 49].

### 3.3 Oxygen, Iron, and Redox State as Rapid Modifiers of RNA Oxidation and Demethylation Capacity

Because Fe(II)/2OG‐dependent dioxygenases require reduced iron and molecular oxygen, sensitivity to hypoxia, iron limitation, and oxidative shifts has been expected to arise as a direct property of the catalytic mechanism rather than as an evolved stress program. In practical terms, these enzymes depend on iron being in the correct reduced state and on oxygen being available for the oxidation reaction. Oxidative stress or altered redox buffering can therefore affect catalysis by changing iron chemistry or by changing the balance of competing metabolites and cofactors. This logic has extended beyond mRNA demethylation. In mitochondria, the formation of 5‐formylcytidine (f5C) at the wobble position of mt‐tRNAMet has been produced through a two‐step pathway in which NSUN3 installs m5C, and ALKBH1 oxidizes that methyl group to f5C, thereby placing a dioxygenase reaction directly within an RNA modification circuit that is functionally tied to mitochondrial translation and metabolic plasticity [74, 75]. In the nucleus, oxidation of RNA m5C by TET2 has been implicated in chromatin‐associated RNA pathways that influence chromatin state and leukemogenesis, demonstrating that RNA oxidation chemistry can contribute to environmental responsiveness at the interface of metabolism and gene regulation [76]. In these systems, rapid changes in oxygen tension, Fe(II) availability, redox buffering, or competing TCA intermediates have been positioned as plausible proximal regulators of oxidation flux, although the timescale at which bulk RNA readouts shift has remained dependent on RNA turnover and compartmental dynamics [49, 74, 75, 76].

### 3.4 Rapid Physical Cues Acting Through Chemistry and RNA Structure

Fast environmental coupling has not been restricted to metabolite control. Temperature shifts and other acute physical stressors have been associated with rapid remodeling of specific RNA modification landscapes in ways that are consistent with immediate changes in enzyme access to substrates or in RNA structural states. In yeast, heat shock has been shown to induce widespread Pus7p‐mediated pseudouridylation, with >200 inducible sites reported (including many in mRNAs), indicating that acute physical stress can be linked to rapid installation of a non‐methyl modification on RNA [50, 51]. Because pseudouridylation is catalyzed without a known eraser system and because RNA structure is a key determinant of pseudouridine synthase targeting, this response supports the idea that environmental inputs can influence substrate availability or enzyme–RNA engagement; however, it should not be interpreted as proof of instantaneous enzymatic switching at all sites, because co‐transcriptional effects and changes in RNA population composition may also contribute [50, 52]. A physical cue such as heat can change RNA folding, RNA‐protein contacts, or the set of newly made transcripts. Any of these changes can alter which RNA sites are accessible to a pseudouridine synthase, even if the enzyme amount itself does not change.

### 3.5 Working Definition of “Fast” Mechanisms for Hypothesis Generation

Across these examples, “fast coupling” has been supported most strongly when a cue has been shown to alter catalytic feasibility through (i) cofactor/cosubstrate dependence (SAM, acetyl‐CoA, 2OG, oxygen, Fe(II)), (ii) direct competitive inhibition by metabolites (R‐2HG, fumarate, citrate), or (iii) rapid shifts in enzyme–substrate engagement under acute physical stress (heat shock–induced pseudouridylation). Importantly, these mechanisms have been most useful as early hypothesis generators for time‐resolved datasets, because they constrain plausible upstream inputs even when mark changes are detected on delayed timescales due to RNA turnover and measurement limits.

## 4 Intermediate Coupling: Signaling, Post‐Translational Modifications, and Relocalization of the Machinery

Intermediate responses most often arise when cue‐triggered signaling reconfigures pre‐existing writers, erasers, readers, or accessory factors, rather than immediately changing metabolites or slowly changing protein abundance. PTMs provide a central interface in this time window because they can alter catalytic output, substrate access, complex assembly, and subcellular localization on the timescale of signaling cascades [33, 34]. PTMs are reversible chemical marks added to proteins after synthesis. These marks can change an enzyme's activity, binding partners, localization signals, or stability, thereby converting a stress‐activated signaling pathway into altered RNA modification or decoding. In practice, phosphorylation, SUMOylation, ubiquitination, acetylation, and O‐GlcNAcylation have been repeatedly documented on core regulators of m6A and on selected readers, and modulation of these PTMs has been linked to environmental cues such as genotoxic stress, hypoxia, oxidative stress, inflammatory cytokines, and nutrient state [25, 33, 34, 53, 54].

### 4.1 Kinase‐Driven Phosphorylation as a Rapid Gating Step

Genotoxic stress has provided some of the clearest causal chains from environmental insult to machinery reprogramming within minutes to hours. In response to DNA double‐strand breaks, METTL3 has been reported to be activated by ATM‐dependent phosphorylation (S43), followed by relocation of phosphorylated METTL3 to damage sites where m6A is installed on damage‐associated RNAs, and YTHDC1 is recruited, thereby supporting homologous recombination repair [53]. YTHDF1 illustrates a parallel reader mechanism: after irradiation, ATR‐dependent phosphorylation at S182 promotes nuclear retention by inhibiting exportin 1–mediated nuclear export, enabling splicing control of DNA damage response transcripts and increasing radioresistance [54]. Earlier work has also shown that m6A writers are recruited to ultraviolet‐induced DNA damage sites within minutes and that local m6A deposition contributes to DNA damage responses, reinforcing that chromatin lesions can be coupled to m6A machinery through fast relocalization programs [25].

Phosphorylation‐dependent control has also been connected to extracellular signaling environments that converge on MAPK pathways. ERK signaling has been identified as a positive regulator of m6A deposition, and phosphorylation of METTL3 (S43/S50/S525) and WTAP (S306/S341) has been shown to promote stabilization of the writer complex through USP5‐mediated deubiquitination, thereby increasing m6A levels and altering fate decisions in stem cells and cancer models [55]. In this setting, signal‐dependent phosphorylation has been positioned as an upstream “permission step” that increases the effective lifetime and activity of the writer machinery without requiring new synthesis of core components [55]. Collectively, these examples have supported a mechanistic model in which kinase cascades convert environmental inputs into rapid changes in machinery state by switching catalytic competence and/or localization, with transcriptome‐scale consequences emerging as RNAs are processed and renewed [25, 53, 54, 55].

### 4.2 SUMOylation and Ubiquitin Circuitry in Stress‐Dependent Tuning of Erasers and Readers

Oxidative stress has been shown to modulate demethylase function through combined phosphorylation and SUMOylation events that alter substrate accessibility. Reactive oxygen species (ROS) have been reported to activate ERK/JNK signaling, leading to phosphorylation of ALKBH5 (S87/S325) and subsequent SUMOylation (including K86/K321), which inhibits demethylase activity by blocking substrate access and results in increased global mRNA m6A and enhanced expression of DNA repair programs [56]. In this case, a cue‐responsive kinase axis has been placed upstream of a PTM cascade on an eraser, and the coupling has been argued to occur on a timescale compatible with rapid stress adaptation [56].

Hypoxia has been shown to modulate reader function through SUMOylation without requiring changes in reader abundance. SUMOylation of YTHDF2 (major site K571) has been reported to be induced by hypoxia and reduced by oxidative stress, and increased SUMOylation has been associated with higher binding affinity to m6A‐modified mRNAs and enhanced decay capacity, thereby reshaping gene expression outputs in cancer‐relevant contexts [57]. Because a single PTM can rapidly alter binding affinity and decay engagement, this mechanism fits the minutes‐to‐hours response window even when downstream transcript‐level readouts appear later [57].

Ubiquitin‐dependent control has been repeatedly implicated as a companion layer that determines whether signaling‐induced activation is sustained or transient. In the ERK–METTL3 axis, USP5‐dependent deubiquitination has been demonstrated to stabilize phosphorylated writer components, thereby linking phosphorylation to protein lifetime and extending the duration of altered m6A deposition beyond the initiating signal [55]. More broadly, PTM‐focused syntheses have emphasized that ubiquitination and deubiquitination frequently act as “duration setters” for environmental responses by regulating turnover of writers/erasers/readers and by reshaping their interactomes [33, 34].

### 4.3 Acetylation and O‐GlcNAcylation as Interfaces to Inflammatory and Nutrient Environments

Inflammatory milieus have been linked to rapid re‐partitioning of writer function through acetylation‐dependent control of subcellular localization. METTL3 acetylation status has been shown to modulate nuclear versus cytosolic distribution, and an IL‐6–associated positive feedback loop has been reported in which IL‐6 signaling promotes METTL3 deacetylation and nuclear translocation, enabling increased nuclear m6A output and supporting metastatic programs in breast cancer models [58]. This mechanism has provided an explicit example in which a cytokine‐defined “environment” is coupled to the writer through reversible lysine acetylation and compartment switching, rather than through slow changes in METTL3 abundance [58].

Nutrient state has been linked to reader and writer regulation through O‐GlcNAcylation, a modification that is commonly treated as sensitive to hexosamine pathway flux and is experimentally tunable on short timescales. O‐GlcNAcylation of YTHDF1 has been shown to increase its cytosolic fraction by enhancing CRM1 binding and nuclear export, and modulation of O‐GlcNAc levels (e.g., through OGA/OGT perturbation and glucose/O‐GlcNAc enrichment conditions) has been used to shift YTHDF1 localization and downstream target expression [59]. O‐GlcNAcylation of METTL3 has also been reported, and enhanced stability and oncogenic output have been linked to this modification in hepatocellular carcinoma models, supporting the plausibility that nutrient‐coupled glycosylation can tune writer abundance and/or activity on intermediate timescales when O‐GlcNAc flux is altered [60]. Together, these studies have supported the view that PTMs associated with metabolic signaling can act as rapid “routing controls” that determine where and how the machinery engages RNA substrates [59, 60].

### 4.4 Relocalization and Condensate‐Gated Access to Marked RNAs

Environmental stress has also been connected to rapid reorganization of RNA–protein assemblies that gate access to substrates and alter how marks are decoded. Stress granules and related condensates can be thought of as temporary RNA–protein assemblies that form during stress and concentrate selected RNAs and binding proteins. By changing which transcripts and readers occupy the same compartment, these assemblies can alter RNA fate without requiring a global change in modification abundance. In response to oxidative stress, additional m6A methylation has been reported in the 5′ vicinity of the coding sequence of selected transcripts, and triaging from the translating pool to stress granules has been facilitated, with selective recognition by YTHDF3 being implicated in this routing [63]. Because stress granules can assemble within minutes and transcript routing can change without global remodeling of all marks, we treat condensate‐associated gating as a plausible intermediate mechanism between direct enzymatic chemistry and slower abundance changes [38, 63]. At the same time, stress granule partitioning depends strongly on general RNA features and translation status, and direct tests detect only limited contributions of m6A to bulk partitioning; condensate‐gated decoding should therefore be treated as context‐dependent rather than universal [62].

### 4.5 Working Definition of “Intermediate” Mechanisms for Hypothesis Generation

Across systems, intermediate coupling has been most strongly supported when a defined environmental cue has been shown to modulate a proximal machinery property via (i) kinase‐dependent phosphorylation and relocalization (ATM/ATR/ERK examples), (ii) SUMOylation‐dependent tuning of demethylase or reader function under ROS or hypoxia, (iii) ubiquitin circuitry that stabilizes or extinguishes signal‐activated machinery, and (iv) acetylation/O‐GlcNAcylation that reroutes machinery localization in inflammatory or nutrient‐linked contexts. These mechanisms have provided experimentally tractable checkpoints—often reversible by pathway inhibitors or PTM perturbation—that can be used to discriminate among candidate upstream sensors when modification changes are detected on the minutes‐to‐hours timescale. However, reader relocalization, condensate‐associated decoding, and individual PTM routes should be treated as context‐dependent until the cue‐specific machinery change and RNA‐level outcome have been linked in the same system.

## 5 Slow Coupling: Transcriptional, Translational, and Proteostatic Control of Machinery Abundance and Specificity

Slow environmental coupling refers to mechanisms that reshape the abundance, stability, isoform usage, or compartmental deployment of epitranscriptomic machinery over hours to days. These mechanisms are organized here into four main routes: transcriptional regulation, sustained stress‐response programs, proteostatic control, and isoform or compartment rewiring. Chronic exposure studies are discussed separately because they provide environmentally relevant contexts but often vary in causal resolution. Causal support is considered strongest when perturbing the upstream cue‐response pathway or machinery interface reduces both the regulator change and the downstream modification phenotype [2, 7].

### 5.1 Transcriptional Induction and Repression of Core Regulators by Cue‐Activated Transcription Factors

Oxygen limitation has been linked to slow coupling through transcriptional control of demethylases. Under hypoxia, HIF‐dependent induction of ALKBH5 changes eraser abundance, and m6A demethylation of defined transcripts connects this response to hypoxia‐adaptive phenotypes [26]. Because demethylase induction requires transcription and translation, this route has been treated as intrinsically slow even when downstream effects on specific transcripts can emerge quickly once the new enzyme has accumulated [26]. Inflammatory environments have been shown to reconfigure the writer complex at the level of expression and assembly propensity. NF‐κB p65 transcriptionally regulates WTAP expression, and WTAP upregulation is associated with increased global m6A in hyperinflammatory states; myeloid WTAP deficiency attenuates inflammatory disease models [64]. In this framework, environmental inflammatory cues have been linked to increased writer‐complex assembly in nuclear speckles via a WTAP‐dependent mechanism, implying that specificity and output can be altered not only by changing protein levels but also by changing the effective concentration of assembled machinery [64].

Endotoxin exposure has been shown to drive slower coupling that combines expression changes with redistribution of the writer. In lung endothelial cells, global m6A levels have been reported to increase after LPS treatment alongside upregulation and nuclear translocation of METTL3, while levels of major demethylases have been reported to remain largely unchanged, supporting a model in which sustained inflammatory signaling increases methylation capacity primarily by elevating the nuclear writer pool [65]. In this setting, transcriptome‐wide shifts in m6A site distribution have been described after LPS exposure, consistent with a slow remodeling of deposition patterns as new transcripts are synthesized and processed under altered writer availability and localization [65].

### 5.2 Stress‐Response Programs That Alter Machinery Abundance During Metabolic and Proteotoxic Environments

Nutrient limitation has been linked to slow coupling through coordinated remodeling of the m6A pathway during autophagy induction. m6A‐dependent control of autophagy‐related translation has been reported during nutrient deficiency, and YTHDF3‐dependent translation of FOXO3 has been shown to promote autophagy flux, with loss of METTL3 impairing this response, indicating that sustained nutrient stress can be routed through writer and reader availability to reshape selective translation programs over hours [78]. Although upstream sensing in nutrient deficiency is typically initiated by rapid kinase signaling, the persistence and scale of epitranscriptomic reprogramming in this context have been compatible with slow rebalancing of RNA production, translation demand, and machinery engagement [78].

Proteotoxic stress has also been connected to slow coupling through unfolded‐protein response contexts. In a trophoblast ER‐stress model induced by thapsigargin, METTL3 expression and global m6A levels have been reported to increase, whereas METTL14 and FTO levels have been reported to remain comparatively unchanged, suggesting that ER stress can selectively elevate writer abundance and thereby bias the modification landscape during prolonged stress adaptation [79]. Because ER stress evolves over hours and engages transcriptional remodeling, this route represents a slow mechanism by which intracellular proteostatic and redox states alter epitranscriptomic capacity [79].

### 5.3 Proteostatic Control as a Slow, Cue‐Sensitive Dial on Machinery Dose and Mark Output

Protein quality‐control systems set the duration and magnitude of epitranscriptomic responses by controlling turnover of writer and non‐m6A machinery components. Here, proteostatic control means that the cell changes how long a writer, eraser, or reader protein persists. For example, ubiquitination can mark a protein for proteasomal degradation, lowering the available machinery dose over time. METTL14 has been shown to undergo STUB1‐mediated ubiquitination and degradation, and METTL3 has been reported to protect METTL14 from this degradation, thereby maintaining m6A homeostasis; in this context, altered E3‐ligase pressure has been shown to change total m6A levels, illustrating that proteostasis can serve as a slow “dose controller” for methylation capacity [66]. Because ubiquitin‐mediated turnover typically integrates upstream stress and signaling inputs over time, proteostatic regulation has been placed within slow coupling even when upstream modifications that trigger ubiquitination occur earlier [66].

Comparable logic has been extended beyond m6A. During hepatocyte ferroptosis, NSUN2 protein levels have been reported to decrease via STUB1‐mediated ubiquitination and degradation, leading to reduced m5C methylation of Gpx4 mRNA and promotion of ferroptotic phenotypes, supporting a mechanism in which oxidative lipid stress is converted into altered writer abundance for a non‐m6A mark over an hours‐scale window [67]. This example has been notable because it links a defined environmental stress modality (iron‐dependent lipid peroxidation) to selective loss of an RNA‐modifying enzyme through proteasomal control, rather than through broad transcriptional repression [67].

Longer‐lived stress states have also been reported to erode writer abundance via dedicated E3 ligases. In senescence models, PRKN (Parkin) has been identified as an E3 ligase that promotes proteasomal degradation of METTL3 through K48‐linked ubiquitination, with reduced m6A and altered expression of senescence‐relevant genes being reported downstream, indicating that chronic stress states can be coupled to global methylation capacity via regulated writer turnover [68].

### 5.4 Isoform Switching and Compartment Rewiring as Slow Determinants of Specificity

Cue‐dependent isoform usage has provided an additional slow mechanism for changing machinery function without altering catalytic domains. Different isoforms of the same RNA‐modifying enzyme can occupy different compartments or interact with different RNA–protein partners. Thus, an environmental cue can change substrate access and pathway engagement by changing which isoform is produced, even when the catalytic activity of the enzyme is not fundamentally altered. For RNA editing, the interferon‐inducible ADAR1p150 isoform has been distinguished from the predominantly nuclear ADAR1p110 isoform, and IFN exposure has been used to rewire the ADAR1 interaction landscape, supporting a model in which inflammatory cues modulate editing capacity and substrate engagement by shifting isoform abundance and compartmental context over hours [69]. Under this view, environmental nucleic acid stress is buffered not only by catalytic editing chemistry but also by slow, cytokine‐driven reallocation of the editor to cytoplasmic ribonucleoprotein environments where dsRNA sensing is engaged [69].

### 5.5 Chronic E2nvironmental Exposures: Associative and Partially Mechanistic Evidence

Chronic exposure studies are important for environmental relevance, but their mechanistic strength varies. We therefore use these examples to illustrate exposure‐linked remodeling of epitranscriptomic regulators while distinguishing direct cue‐to‐machinery evidence from associative changes in regulator expression or modification abundance. Environmentally relevant toxicants have been shown to reprogram the epitranscriptome on timescales consistent with slow coupling. During microcystin‐LR exposure, increased m6A levels have been reported after multi‐day exposure windows, and changes in writer/eraser/reader expression have been described alongside ALKBH5‐dependent effects on metabolic reprogramming [80]. This provides partially mechanistic support for toxin‐linked m6A remodeling, but the upstream route from toxin exposure to altered machinery state remains less resolved than in systems where the cue‐response pathway or machinery interface is directly perturbed [80]. In this setting, the effective “sensor” is best interpreted as toxin‐triggered cellular stress and metabolic imbalance, rather than as direct toxin detection by the demethylase machinery [80]. Metal exposure has likewise been associated with slow remodeling of m6A control layers. In cadmium‐treated lung cells, altered m6A phenotypes have been reported together with engagement of NF‐κB–linked stress responses, and a mechanistic link has been proposed between cadmium‐induced oxidative stress and m6A regulatory protein [81]. Because this evidence does not yet establish a complete cue‐to‐machinery‐to‐mark chain, it is treated here as exposure‐linked, partially mechanistic support for slow coupling through stress‐programmed machinery regulation rather than direct catalytic inhibition alone.

Together, these slow‐coupling examples illustrate that delayed epitranscriptomic responses can arise through several mechanistically distinct routes. Transcriptional induction, proteostatic turnover, and isoform rewiring provide stronger machinery‐level entry points for causal testing, whereas chronic exposure studies are most informative when they are paired with perturbations that identify the upstream stress pathway and the affected writer, eraser, or reader interface.

## 6 A Kinetics‐to‐Sensors Framework for Causal Inference

**FIGURE 3:**
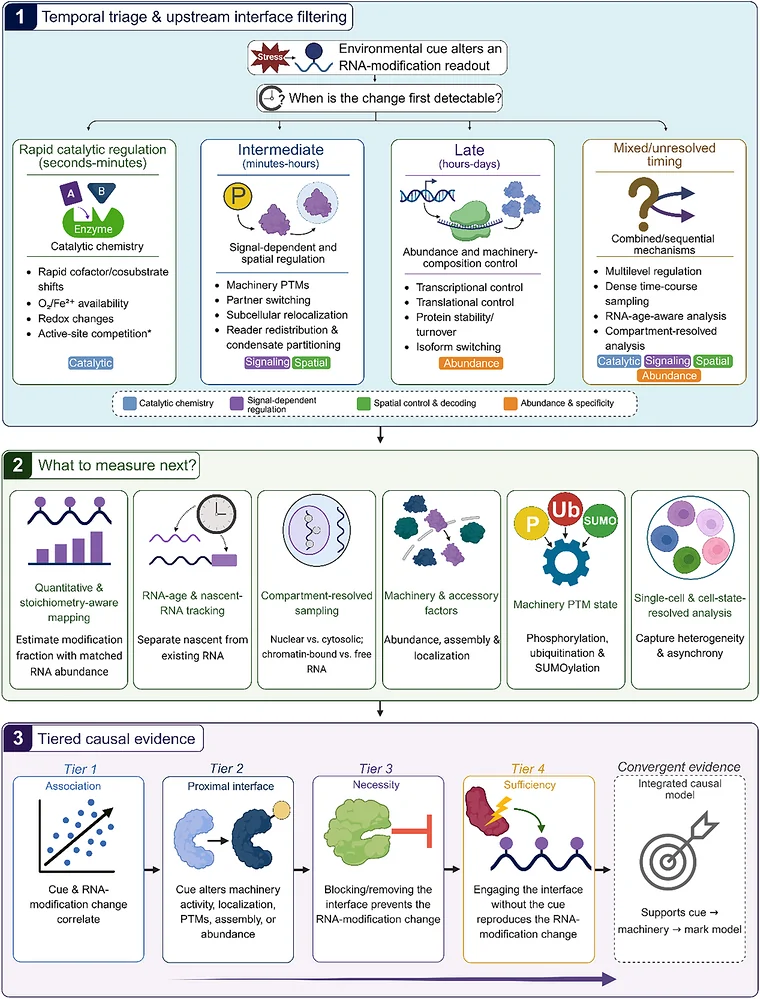
Decision workflow for testing cue‐to‐mark causality. The workflow starts from an environmental cue associated with an RNA‐modification change and uses response timing to prioritize candidate upstream interfaces. Rapid responses point first to catalytic chemistry, including cofactor or cosubstrate availability, oxygen or iron availability, redox state, or active‐site competition. Intermediate responses point to PTMs, partner switching, relocalization, reader redistribution, or condensate partitioning. Late responses point to transcriptional or translational regulation, protein turnover, or isoform switching. Mixed timing may indicate combined or sequential mechanisms and should prompt RNA‐age‐aware, compartment‐resolved, or machinery‐state measurements. Timing is approximate and context‐dependent; it prioritizes mechanisms but does not establish causality. Measurements should match the proposed mechanism and may include stoichiometry‐aware mapping with RNA‐abundance controls, nascent‐RNA tracking, compartment‐resolved sampling, direct or isoform‐resolved detection, machinery‐state measurements, and single‐cell or cell‐state‐resolved analysis. Causal confidence increases through temporal association, proximal‐interface support, necessity testing, sufficiency or complementary causal testing, and integration into a cue‐to‐interface‐to‐machinery‐to‐RNA‐modification model. PTM, post‐translational modification; RNA‐modification stoichiometry, fraction of RNA molecules carrying a modification at a defined site or RNA population; condensate partitioning, preferential enrichment or exclusion within biomolecular condensates.

A central premise of this Review is that response speed can help narrow the set of plausible upstream mechanisms. Biochemical modulation, signaling‐dependent rewiring, and abundance‐control programs act on different kinetic regimes, so the timing of a modification change can guide hypothesis generation [2, 7, 13, 35]. However, timing is not sufficient on its own. Bulk RNA measurements can lag behind machinery regulation because RNA synthesis, RNA decay, compartment mixing, and assay‐specific biases can all reshape apparent modification levels [13, 35, 37]. Therefore, kinetics is used here as a hypothesis filter, not as a stand‐alone mechanistic verdict. This logic is currently best supported for m^6^A, where quantitative tools, perturbation systems, and machinery annotations are comparatively mature. For non‐m^6^A marks, the same kinetic reasoning should be applied more cautiously and adapted to the chemistry, reversibility, abundance, and measurement constraints of each modification. The decision workflow in Figure 3 summarizes how response timing, measurement principles, and perturbation tests can be combined to move from an observed cue‐associated RNA‐modification change to a causal cue‐to‐interface‐to‐machinery model.

### 6.1 Step 1: Time‐Resolved Sampling Should be Designed to Separate Chemistry From Population Turnover

Time courses have been recommended to include early windows (minutes) and later windows (hours to days), because fast coupling can be missed when only endpoint measurements are collected [7, 35]. The value of early sampling has been illustrated by localized m6A deposition at UV‐induced DNA damage sites within minutes, which has shown that acute physical perturbations can be coupled to RNA marking far faster than canonical transcriptional remodeling [25]. Fast redistribution of marking has also been supported under heat shock, where preferential methylation of 5′ UTR regions of newly transcribed stress mRNAs has been reported together with reader relocalization, implying that coupling can occur during the first wave of stress adaptation [24]. Conversely, transient or co‐transcriptional marking has been shown to be under‐detected in steady‐state measurements, and nascent/transient m6A profiling has been used to reveal short‐lived modification events that would be blurred in bulk RNA maps [39, 82]. Therefore, inference has been strengthened when kinetic sampling has been paired with strategies that explicitly track newly synthesized RNA (or newly incorporated modified nucleosides), rather than relying solely on steady‐state poly(A)+ pools [37, 39, 40].

Quantitative MS‐based approaches have been positioned as particularly informative for kinetics when global nucleoside pools are being perturbed, because turnover of modified ribonucleosides can be measured directly and decoupled from mapping noise [37, 40]. A ^13^C‐labeling strategy (^13^C‐dynamods) has been developed to quantify turnover of multiple modified ribonucleosides in newly transcribed RNA, thereby enabling the timing of modification accrual to be constrained experimentally under nonstationary conditions [37]. Such approaches have been recommended as complements to sequencing‐based mapping, because many widely used mapping methods remain enrichment‐based and not inherently stoichiometric [35, 37].

### 6.2 Step 2: Measurement Strategy Should be Matched to the Kinetic Question

Because “change in modification” can refer to distinct observables (site appearance, occupancy shifts at existing sites, or shifts in the modified fraction of transcript isoforms), assay choice has been encouraged to be aligned with the specific kinetic hypothesis being tested [13, 35, 37]. Recent methodological reviews have emphasized that causal claims about upstream sensing are most robust when at least one quantitative axis is included (site‐specific stoichiometry, modification turnover, or isoform‐resolved modified fractions), rather than relying exclusively on peak calling or antibody enrichment intensity [35, 37]. Several measurement principles are especially important for kinetics‐aware inference. First, if the question is whether modification occupancy changes, stoichiometry‐aware approaches are needed rather than peak intensity alone. MAZTER‐seq and m6A‐LAIC‐seq illustrate this principle by estimating site‐level or isoform‐level methylated fractions at defined subsets of m6A sites [83, 84]. Second, if antibody enrichment is a concern, antibody‐independent approaches can provide useful orthogonal evidence; DART‐seq is one example for relative m6A tracking across perturbations [85]. Third, if the question involves molecule‐to‐molecule heterogeneity, isoform context, or direct detection, nanopore direct RNA sequencing and related single‐molecule approaches may be informative, but their modification calls require benchmarking and orthogonal validation [72, 86]. Fourth, if bulk kinetics may reflect asynchronous responses across cells, single‐cell or state‐resolved designs should be considered. scDART‐seq illustrates how cell‐to‐cell heterogeneity in m6A can make a population‐average change appear slower or smoother than the underlying cellular response [87]. Thus, these methods are used here as examples of broader measurement principles: quantitative interpretation, isoform or RNA‐age resolution, antibody‐independent validation, direct detection, and heterogeneity‐aware analysis summarized in Table 3 [13, 35, 37]. Because many of these approaches were developed first for m^6^A, equivalent inference for non‐m^6^A marks requires mark‐specific validation rather than direct transfer of m^6^A‐based assumptions.

**TABLE 3:** Kinetics‐aware measurement and validation toolkit. Recommended experimental readouts matched to the kinetic hypothesis (chemistry vs. routing vs. abundance). Includes what each method can and cannot conclude when used for cue sensing claims.

Kinetic question	Best‐fit primary measurements	What it resolves	Key controls to avoid false “sensing”	Key references
**Is the change chemistry‐driven (fast proximal)?**	Metabolomics (SAM/SAH, 2OG, fumarate, etc.); enzyme activity proxies; MS nucleoside turnover	Proximal feasibility vs. downstream lag	RNA age tracking; compartment control	[31, 32, 37]
**Is it co‐transcriptional / transient marking?**	Nascent RNA profiling; chromatin‐associated RNA; time‐resolved mapping	Early deposition events disappear in steady state	Compare nascent vs. poly(A)+; transcription block experiments	[39, 82]
**Are maps stoichiometric enough for kinetics?**	Quantitative/stoichiometry‐aware mapping + MS	Fraction modified vs. peak strength	Spike‐ins; orthogonal methods; replicate‐rich sampling	[35, 70]
**Track dynamic m6A without antibodies**	DART‐seq	Relative changes across perturbations	Validate editing background; orthogonal mapping	[85]
**Site‐limited stoichiometry subset**	MAZTER‐seq	Quantitative stoichiometry at eligible motifs	Motif coverage limitations; RNA structure controls	[83]
**Isoform‐resolved methylated fraction**	m6A‐LAIC‐seq	Modified vs. unmodified isoform fractions	Normalize for isoform abundance changes	[84]
**Single‐molecule & multi‐mark potential**	Nanopore direct RNA + benchmarking	Heterogeneity; multi‐feature inference	Use benchmarked callers; cross‐method triangulation	[71, 72]
**Does “slow” bulk change reflect heterogeneity?**	Single‐cell m6A approaches (e.g., scDART‐seq)	Cell‐state mixing vs true slow chemistry	Sorted populations; lineage/state markers	[87]
**Is condensate gating causal?**	Imaging + partitioning assays + functional disruption	Reader routing and functional dependency	Control for transcript length/translation; condensate disruption controls	[38, 61, 62]

### 6.3 Step 3: Upstream Sensors Should be Shortlisted by Timescale, Then Tested With Discriminating Perturbations

We base the practical decision logic on the observation that several cue‐to‐mark mechanisms cluster into three kinetic regimes: direct biochemical interfaces with fast proximal potential, signaling/PTM and localization control (minutes–hours), and abundance/proteostasis programs (hours–days) [2, 7, 33, 35]. The first category refers to the earliest possible effect on catalytic feasibility or substrate access once the relevant chemical or physical input is present; it does not imply that metabolite accumulation or transcriptome‐wide RNA readouts always occur within seconds to minutes. Representative evidence has been used to anchor each regime.

#### 6.3.1 Fast‐Proximal Mechanisms: Chemical Feasibility and Competitive Inhibition

Rapid coupling has been considered most compatible with mechanisms in which catalytic feasibility is altered directly by substrate/cofactor pools or by competitive metabolites, even if transcriptome‐wide readouts emerge later due to metabolite accumulation, compartmental access, or RNA turnover [2, 7, 35, 40]. A SAM‐sensitive regulatory circuit has been demonstrated for METTL16 under methionine starvation/SAM depletion through altered engagement with MAT2A RNA hairpins, providing a well‐defined example in which a metabolite pool can be coupled directly to writer behavior [40, 41, 42]. Competitive metabolite inhibition of erasers has been supported by R‐2HG inhibition of FTO, linking a single metabolite to increased m6A levels and downstream gene‐expression programs [48]. More recently, fumarate accumulation has been shown to rewire RNA methylation in renal cancer contexts in a manner consistent with metabolite pressure on demethylation capacity, providing an additional example in which metabolic state can act upstream of epitranscriptomic output without requiring primary changes in machinery abundance [49]. In both cases, the biochemical interface is direct, but the cellular manifestation may occur over longer intervals because the inhibitory metabolite must accumulate and reach the relevant enzyme pool. In this regime, rescue or mimicry experiments (metabolite supplementation, donor depletion, or competitive inhibitors) have been treated as discriminating tests because proximal chemistry is being perturbed directly, and these approaches have been central to mechanistic resolution in the examples above [41, 48, 49, 53].

#### 6.3.2 Minutes–Hours: PTMs, Partner Switching, and Relocalization

Intermediate coupling is strongest when the causal sequence is explicit: the cue activates signaling, signaling modifies a writer, eraser, reader, or accessory factor, this changes activity, localization, binding, or stability, and the RNA mark or RNA fate output changes accordingly [33]. ATM‐dependent phosphorylation and recruitment of METTL3 to DSB sites has been shown to couple genotoxic stress to local m6A deposition and DNA repair factor recruitment, providing a clear cue‐to‐machinery chain within an intermediate window [53]. ROS‐induced PTM cascades on ALKBH5 (phosphorylation/SUMOylation) have been shown to inhibit demethylase function and increase global m6A during oxidative stress, supporting a direct route from redox stress to eraser activity modulation [56]. ATR‐dependent phosphorylation of YTHDF1 with inhibited nuclear export has been shown after irradiation, further illustrating that reader localization can be rapidly rewired by damage‐responsive signaling [54]. In this regime, discriminating perturbations have been most effectively implemented by pathway inhibition (e.g., ATM/ATR/MAPK axis inhibition), PTM‐pathway interference, or forced localization mutants, because the proximal control point is PTM and routing rather than metabolite chemistry or de novo synthesis [33, 38, 54, 56].

#### 6.3.3 Hours–Days: Transcriptional, Translational, and Proteostatic Remodeling

Slow coupling has been considered most compatible with programs that change the abundance, isoform usage, or stability of machinery components, as has been discussed in broader reviews of environmentally responsive RNA modification programs [2, 7, 35]. In this regime, dependence on transcription/translation has often been tested experimentally, and mechanistic strength has been increased when mark changes have tracked with measured changes in regulator abundance and when upstream stress programs have been shown to be required [2, 7, 35]. Because slow coupling can be superimposed on faster regulation (e.g., early PTMs followed by later abundance shifts), mixed‐kinetic models have been recommended to be evaluated explicitly rather than assumed away [7, 33, 35].

### 6.4 Step 4: Condensate‐Gated Decoding Should be Treated as a Testable Branch, not a Default Explanation

Stress‐induced condensates have been emphasized as plausible intermediate mechanisms because assemblies such as stress granules can form rapidly and can re‐partition RNAs and RNA‐binding proteins within minutes [63]. m6A‐dependent triaging of transcripts to stress granules has been proposed under oxidative stress with YTHDF3 involvement, supporting a model in which environmental stress can be coupled to mark interpretation through condensate routing [63]. However, limited effects of m6A on bulk mRNA partitioning into stress granules have been reported in direct tests, indicating that condensate gating is context‐dependent and should be validated rather than assumed [62]. Therefore, condensate‐based hypotheses should be tested through reader relocalization, binding requirements, and functional consequences of condensate disruption, rather than through co‐occurrence of stress granules and mark changes alone [62, 63].

### 6.5 Step 5: A Prioritized, Kinetics‐Aware Experimental Checklist Should be Applied

For kinetics‐to‐sensors inference to be reproducible, the experimental checklist should be treated as prioritized rather than uniform: some elements are essential for any cue‐to‐mark claim, whereas others are context‐dependent controls that strengthen causal interpretation. At minimum, studies should measure the RNA modification change together with transcript abundance and should test whether the proposed machinery component or coupling interface is altered by the cue. When response speed is central to the claim, RNA‐age or turnover‐aware measurements become essential, because apparent modification changes can otherwise reflect altered RNA synthesis or decay rather than altered marking chemistry [35, 37, 40]. Compartment‐aware sampling should be prioritized when relocalization, chromatin‐associated marking, or cytoplasmic reader redistribution is proposed, because whole‐cell lysates can dilute decisive signals [38, 54, 56]. Orthogonal or stoichiometry‐aware methods are strongly recommended when enrichment‐based mapping is used, but the specific method can be adapted to the mark and system [13, 37, 72, 83, 84, 85]. Single‐cell or state‐resolved designs are most important when heterogeneous or asynchronous responses are likely, because bulk kinetics can be distorted by asynchronous cellular responses and by state mixing [87]. Thus, the checklist is intended to provide a graded route to stronger causal inference rather than a fixed experimental template for every RNA modification pathway. This distinction is particularly important for non‐m^6^A marks, where perturbation tools, stoichiometry‐aware assays, and reader/eraser annotations may be less mature, and where the machinery may not follow the canonical m^6^A writer–eraser–reader model.

## 7 Conclusion and Outlook: Key Gaps, Best Practices, and a Roadmap for Cue‐to‐Mark Mechanisms

Studies increasingly document environmental responsiveness across RNA modification pathways. However, mechanistic attribution remains uneven because many cue‐to‐mark chains are still inferred from endpoint maps rather than tested through proximal control points on writers, erasers, and readers [2, 19, 23]. As a result, “environmental epitranscriptomics” has been described as a rapidly expanding evidence base that is still limited by fragmentation across exposure science, metabolism, signaling, and RNA biochemistry [19, 23]. Regulation is also mark‐specific: non‐m6A modifications can differ substantially in stoichiometry, deposition timing, reversibility or chemical interconversion, and decoding logic, which prevents direct generalization from m6A alone [13]. For these reasons, we argue that the field now needs to move from descriptive catalogs of stress‐associated modification changes toward testable models that connect defined cues to defined proximal control points on modification machinery [2, 13, 19, 23]. A clearer explanation of why RNA modifications participate in these responses is also needed: their value lies in linking environmental information to RNA fate decisions with a speed, selectivity, and compartmental resolution that transcriptional regulation alone cannot always provide.

Several mechanistic gaps have remained particularly constraining, but they are not equally limiting for causal inference. The most immediate priority is to define proximal cue‐to‐machinery interfaces, because many studies still infer sensing from endpoint modification maps rather than from measured changes in writer, eraser, reader, or accessory‐factor behavior. Within this priority, metabolite–enzyme coupling remains a central gap, despite strong biochemical expectation that donor, cosubstrate, and product pools can rapidly gate catalytic flux [31, 32]. A second high‐priority gap is the temporal ordering of PTM‐based regulation, because cue specificity and the distinction between trigger PTMs and duration‐setting PTMs often remain incompletely mapped, particularly outside cancer‐centric contexts [33, 34]. A third priority is to treat decoding as an active regulatory layer, because reader engagement can be rapidly altered by localization, partner switching, and condensate partitioning, even when installation chemistry is unchanged [2, 33]. Beyond these immediate causal gaps, exposure‐relevant systems need deeper mechanistic integration, because they provide controlled perturbations for distinguishing direct chemical interference from slower stress‐program reconfiguration [19, 23]. Finally, cross‐talk among RNA modifications remains an important but later‐stage challenge, because it requires quantitative, multi‐mark measurements synchronized in time and matched to RNA age and compartment [2, 13].

Methodological constraints have been repeatedly identified as the main reason why mechanism has lagged behind mapping. Quantitative interpretation has remained challenging because many commonly used mapping approaches are not inherently stoichiometric, and apparent “mark changes” can be driven by RNA turnover, transcript isoform shifts, or cell‐state mixing rather than by altered enzymatic flux on existing RNA molecules [13, 35]. Therefore, we consider mechanistic inference strongest when studies measure at least one quantitative axis, such as site‐level stoichiometry, RNA‐age‐resolved dynamics, or direct modified nucleoside turnover, alongside positional maps and expression changes [35]. Single‐cell heterogeneity has further complicated kinetic reasoning in bulk samples, because population‐average trajectories can be generated by asynchronous switching across subpopulations rather than by slow chemistry within individual cells [87]. Consequently, heterogeneity‐aware designs have been positioned as critical when response speed is being used to constrain upstream sensors [35, 87].

A practical roadmap for establishing cue sensing by epitranscriptomic machinery can be implemented as a graded evidence framework rather than a universal minimum standard. At the strongest level of causal inference, (i) a proximal machinery property should be shown to be altered by the cue (activity, substrate access, complex assembly, localization, PTM state, or abundance) on an interpretable timescale [2, 33, 34]. (ii) Necessity should be tested by asking whether disruption of the proposed coupling interface, such as metabolite rescue/depletion, pathway inhibition, PTM‐site mutation, forced localization, or targeted depletion of the regulator, reduces or abolishes the mark change and the associated RNA fate output [31, 32, 33, 34]. (iii) Sufficiency should be tested when feasible by perturbing the same interface in the absence of the original cue and asking whether this reproduces the mark change with comparable directionality and kinetics [31, 32, 33, 34]. (iv) RNA age and compartment should be controlled when technically possible, because early mark shifts can reflect preferential marking of newly synthesized RNA rather than turnover on mature transcripts, and compartment mixing can conceal decisive relocalization events [13, 35]. These criteria should therefore be viewed as an aspirational route to stronger causal inference, not as a fixed requirement for every RNA modification system. For marks or organisms where perturbation tools and quantitative assays are still limited, partial evidence can still be informative if the level of causal support is stated clearly. Under this framework, confidence increases when proximal control is shown first, and transcriptome‐wide consequences are interpreted second, rather than the reverse [2, 13, 35].

Progress in single‐molecule and benchmarking efforts has begun to reduce the technical barrier to such causal work, although these approaches should still be interpreted with appropriate caution. Nanopore‐based direct RNA sequencing has been increasingly used for integrated views of transcript structure and modification potential, but comparability across algorithms and datasets has been a persistent limitation [35]. Community benchmarking has been introduced to quantify error modes and improve consistency of modification prediction from nanopore signals, thereby supporting more standardized evaluation of modification callers as these platforms become more widely used [72]. However, nanopore‐based modification detection remains dependent on model training, coverage, sequence context, RNA quality, and the availability of appropriate controls. Therefore, nanopore‐derived modification signals should not be treated as stand‐alone evidence for cue‐to‐mark causality. In parallel, single‐molecule models for m6A detection from direct RNA sequencing data have been developed to resolve heterogeneity among RNA molecules from the same transcript, enabling cue effects to be decomposed into shifts in modified fractions rather than being inferred from bulk averages alone [86]. For environmental sensing studies, these approaches are likely to be most powerful when paired with orthogonal validation, such as quantitative mass spectrometry, antibody‐independent mapping, stoichiometry‐aware assays, or targeted biochemical validation of the proposed machinery interface. These advances are expected to be particularly valuable for environmental sensing questions, where partial penetrance and cell‐state stratification are common and where rapid changes can be confined to specific compartments or RNA subpools [19, 72, 86, 87].

Several field‐level directions have been implied by the synthesis presented here. First, cue classes should be treated explicitly as mechanistic priors: cues that immediately perturb metabolism, oxygen tension, or metal/oxidant burden should initially direct attention to chemical gating and competitive inhibition, whereas cues that primarily engage receptor signaling should prompt early testing of PTM, relocalization, and reader‐routing mechanisms before slower abundance programs are invoked [31, 32, 33, 34]. Second, multi‐omics alignment should be prioritized in time‐resolved designs, because metabolomics, phosphoproteomics/PTM profiling, and quantitative proteomics of writers/erasers/readers can identify proximal control points that are not detectable from RNA maps alone [19, 31, 32, 33, 34, 88]. Third, non‐m6A pathways should be incorporated as integral components of environmental sensing models, because the diversity of chemistries and cellular compartmentalization across marks is likely to encode different sensitivity spectra to temperature, redox, nutrients, and toxicants [13]. Fourth, exposure biology and mechanistic RNA biology should be more directly integrated, because recent exposure‐focused reviews have emphasized the potential of RNA modifications as biomarkers and mediators while also highlighting the need for causal resolution before translation can be justified [19, 20, 23].

Finally, this mechanistic framework also has translational relevance, because RNA modification systems are emerging as drug targets. Although therapeutic development remains early for many pathways, researchers increasingly discuss inhibition strategies against selected writers, readers, and erasers, with proof‐of‐concept small‐molecule inhibitors reported for METTL3 and FTO [89, 90, 91]. In this context, defining how environmental and microenvironmental cues alter machinery activity, localization, or abundance may help explain variation in target engagement and therapeutic response. Mechanistic clarity about cue‐to‐machinery coupling is not only expected to improve interpretation of epitranscriptomic datasets, but also to inform when environmental context will modulate target engagement and efficacy for emerging epitranscriptome‐directed therapeutics [89].

**A kinetics‐aware roadmap and minimum‐evidence standard for cue‐to‐mark causality**

**Table:** 

A unified guide that (i) defines what counts as cue sensing by epitranscriptomic machinery and (ii) outlines a minimum‐evidence ladder to establish causal cue—machinery—mark relationships while avoiding common confounders in time‐resolved datasets. **A. Definitions and boundaries** **Environmental cue**: exogenous exposures and endogenous microenvironment constraints that alter cellular chemistry or signaling state (e.g., nutrients, oxygen, oxidants, cytokines, radiation). Sensing (operational): a cue reproducibly alters a proximal machinery property (activity, substrate access, complex assembly, localization, PTM state, or abundance), and this change is consistent with the observed mark shift. **Direct vs indirect coupling**: · **Direct**: cue changes catalytic feasibility or substrate/cofactor availability without intermediate signaling/transcription/translation. · **Indirect**: cue acts through signaling, transcriptional, translational, or proteostatic programs that reconfigure the machinery. **Kinetics as constraint, not proof**: bulk marks can lag due to RNA synthesis/decay, compartment mixing, and non‐stoichiometric mapping outputs. **B. Minimum‐evidence ladder** **1. Proximal change first**: show cue alters a machinery property on an interpretable timescale (activity, binding/access, PTM, localization, or abundance). **2. Necessity**: disrupt the proposed interface (metabolite rescue/depletion, pathway inhibitor, PTM‐site mutation, forced localization, regulator depletion) and show the mark change (and linked RNA fate readout) is lost. **3. Sufficiency**: perturb the same interface without the original cue and reproduce the mark change with comparable directionality/kinetics. **4. Control RNA age and compartment**: include nascent‐RNA or turnover‐aware designs; fractionate where relocalization is proposed. **5. Triangulate quantitation**: include at least one quantitative axis (MS turnover, stoichiometry‐aware mapping, or isoform‐resolved modified fractions) alongside positional maps.

## Funding

The authors have nothing to report.

## Conflicts of Interest

The authors declare no conflicts of interest.

## Data Availability

Data sharing not applicable to this article as no datasets were generated or analysed during the current study.

## References

[1] D. Sordyl , E. Boileau , A. Bernat , et al., “MODOMICS: A Database of RNA Modifications and Related Information. 2025 Update and 20th Anniversary,” Nucleic Acids Research 54 (2025): D219–D225 . 10.1093/nar/gkaf1284 PMC12807697 41277531

[2] S. Delaunay , M. Helm , and M. Frye , “RNA Modifications in Physiology and Disease: Towards Clinical Applications,” Nature Reviews Genetics 25 (2023): 104–122, 10.1038/s41576-023-00645-2. 37714958

[3] K. Karikó , H. Muramatsu , F. A. Welsh , et al., “Incorporation of Pseudouridine Into mRNA Yields Superior Nonimmunogenic Vector With Increased Translational Capacity and Biological Stability,” Molecular Therapy 16 (2008): 1833–1840, 10.1038/mt.2008.200. 18797453 PMC2775451

[4] K. Karikó , M. Buckstein , H. Ni , and D. Weissman , “Suppression of RNA Recognition by Toll‐Like Receptors: The Impact of Nucleoside Modification and the Evolutionary Origin of RNA,” Immunity 23 (2005): 165–175, 10.1016/j.immuni.2005.06.008. 16111635

[5] U. Sahin , K. Karikó , and Ö. Türeci , “mRNA‐Based Therapeutics — Developing a New Class of Drugs,” Nature Reviews Drug Discovery 13 (2014): 759–780, 10.1038/nrd4278. 25233993

[6] E. P. Ahi and Z. Ghasemishahrestani , “Epitranscriptomic Control of Telomere Maintenance,” Molecular Biology Reports 53 (2026): 513, 10.1007/s11033-026-11699-w. 41854934 PMC13002708

[7] J. Cerneckis , G. L. Ming , H. Song , C. He , and Y. Shi , “The Rise of Epitranscriptomics: Recent Developments and Future Directions,” Trends in Pharmacological Sciences 45 (2024): 24–38, 10.1016/j.tips.2023.11.002. 38103979 PMC10843569

[8] M. Khorshid and E. P. Ahi , “RNA Aptamers and Epitranscriptomics: Charting Unexplored Territories in RNA Biology,” Molecular Diagnosis and Therapy 30 (2026): 383–402. 41838348 10.1007/s40291-026-00841-w PMC13171942

[9] L. Qiu , Q. Jing , Y. Li , and J. Han , “RNA Modification: Mechanisms and Therapeutic Targets,” Molecular Biomedicine 4 (2023): 25. 37612540 10.1186/s43556-023-00139-x PMC10447785

[10] X. Jiang , B. Liu , Z. Nie , et al., “The Role of m6A Modification in the Biological Functions and Diseases,” Signal Transduction and Targeted Therapy 6 (2021): 74. 33611339 10.1038/s41392-020-00450-x PMC7897327

[11] K. D. Meyer and S. R. Jaffrey , “The Dynamic Epitranscriptome: N6‐Methyladenosine and Gene Expression Control,” Nature Reviews Molecular Cell Biology 15 (2014): 313–326, 10.1038/nrm3785. 24713629 PMC4393108

[12] K. D. Meyer and S. R. Jaffrey , “Rethinking M 6 A Readers, Writers, and Erasers,” Annual Review of Cell and Developmental Biology 33 (2017): 319–342, 10.1146/annurev-cellbio-100616-060758. PMC5963928 28759256

[13] H. Sun , K. Li , C. Liu , and C. Yi , “Regulation and Functions of Non‐m6A mRNA Modifications,” Nature Reviews Molecular Cell Biology 24 (2023): 714–731, 10.1038/s41580-023-00622-x. 37369853

[14] W. W. Liu , S. Q. Zheng , T. Li , et al., “Multifunctional Nanoparticle‐Mediated Combining Therapy for Human Diseases,” Signal Transduction and Targeted Therapy 9 (2024): 1, 10.1038/s41392-023-01668-1. 38161204 PMC10758001

[15] A. C. Nagaraj , E. P. Ahi , M. L. Änkö , and J. Kaslin , “RNA Signaling in Cellular Plasticity During Homeostasis and Regeneration,” Current Opinion in Genetics & Development 99 (2026): 102478, 10.1016/j.gde.2026.102478. 42054856

[16] E. P. Ahi , “Epitranscriptomics as a Candidate Universal Modulator of Dormancy Transitions,” Ecology & Evolution 16 (2026): 73007. 10.1002/ece3.73007 PMC12867678 41646876

[17] E. P. Ahi , “Bridging Maternal Effects and Epitranscriptomics: A Novel Perspective in Developmental Biology,” Developmental Dynamics 255 (2025): 628–645. 41467365 10.1002/dvdy.70111 PMC13353613

[18] A. Cayir , H. M. Byun , and T. M. Barrow , “Environmental Epitranscriptomics,” Environmental Research 189 (2020): 109885, 10.1016/j.envres.2020.109885. 32979994

[19] S. Wei , H. Y. Tao , Z. Duan , and Y. Wang , “Environmental Exposure, Epitranscriptomic Perturbations, and Human Diseases,” Environmental Science & Technology 59 (2025): 6387–6399. 40126397 10.1021/acs.est.5c00907 PMC11978485

[20] E. P. Ahi and P. Singh , “The Importance of Alternative Splicing in Adaptive Evolution,” Molecular Ecology 31 (2022): 1928–1938. 35094439 10.1111/mec.16377

[21] M. Frapin , L. Quispe , J. Troka , et al., “Density‐Dependent Expression of Epitranscriptomic, Stress and Appetite Regulating Genes in Atlantic Salmon,” Molecular Ecology 35 (2026): 70230, 10.1111/mec.70230. PMC12759207 41482736

[22] E. Malovic , A. Ealy , A. Kanthasamy , and A. G. Kanthasamy , “Emerging Roles of N6‐Methyladenosine (m6A) Epitranscriptomics in Toxicology,” Toxicological Sciences 181 (2021): 13–22, 10.1093/toxsci/kfab021. 33616673 PMC8599717

[23] E. U. Alum , R. I. Ejemot‐Nwadiaro , M. Basajja , D. E. Uti , O. P. C. Ugwu , and P. M. Aja , “Epitranscriptomic Alterations Induced by Environmental Toxins: Implications for RNA Modifications and Disease,” Genes and Environment 47 (2025): 14, 10.1186/s41021-025-00337-9. 40760453 PMC12323242

[24] J. Zhou , J. Wan , X. Gao , X. Zhang , S. R. Jaffrey , and S. B. Qian , “Dynamic m6A mRNA Methylation Directs Translational Control of Heat Shock Response,” Nature 526 (2015): 591–594, 10.1038/nature15377. 26458103 PMC4851248

[25] Y. Xiang , B. Laurent , C. H. Hsu , et al., “RNA M^6^A Methylation Regulates the Ultraviolet‐Induced DNA Damage Response,” Nature 543 (2017): 573–576. 28297716 10.1038/nature21671 PMC5490984

[26] C. Zhang , D. Samanta , H. Lu , et al., “Hypoxia Induces the Breast Cancer Stem Cell Phenotype by HIF‐Dependent and ALKBH5‐Mediated M^6^A‐Demethylation of NANOG mRNA,” PNAS 113 (2016): E2047. 27001847 10.1073/pnas.1602883113 PMC4833258

[27] R. J. Ries , S. Zaccara , P. Klein , et al., “m^6^A Enhances the Phase Separation Potential of mRNA,” Nature 571 (2019): 424–428. 31292544 10.1038/s41586-019-1374-1 PMC6662915

[28] M. Ponzetti , N. Rucci , and S. Falone , “RNA Methylation and Cellular Response to Oxidative Stress‐Promoting Anticancer Agents,” Cell Cycle 22 (2023): 870–905, 10.1080/15384101.2023.2165632. 36648057 PMC10054233

[29] T. Nakahama and Y. Kawahara , “The RNA‐Editing Enzyme ADAR1: A Regulatory Hub That Tunes Multiple dsRNA‐Sensing Pathways,” International Immunology 35 (2023): 123–133, 10.1093/intimm/dxac056. 36469491

[30] S. S. Bhat , M. Paul , and B. D. Gregory , “Epitranscriptomic Modifications in Plant RNAs,” RNA Biology 22 (2025): 1–14, 10.1080/15476286.2025.2515663. PMC12150641 40464679

[31] J. Kim and G. Lee , “Switching From Insulin to Dulaglutide Therapy in Patients With Type 2 Diabetes: A Real‐World Data Study,” Diabetes and Metabolism Research and Review 37 (2021): 3466 . 10.1002/dmrr.3466 33957706

[32] J. M. Thomas , P. J. Batista , and J. L. Meier , “Metabolic Regulation of the Epitranscriptome,” ACS Chemical Biology 14 (2019): 316–324, 10.1021/acschembio.8b00951. 30653309

[33] Y. Lin , P. Lin , Y. Lu , et al., “Post‐Translational Modifications of RNA‐Modifying Proteins in Cellular Dynamics and Disease Progression,” Advanced Science 11 (2024): 2406318, 10.1002/advs.202406318. 39377984 PMC11600222

[34] Y. Wang , Y. Wang , H. Patel , et al., “Epigenetic Modification of m6A Regulator Proteins in Cancer,” Molecular Cancer 22 (2023): 102, 10.1186/s12943-023-01810-1. 37391814 PMC10311752

[35] B. He , Y. Chen , and C. Yi , “Quantitative Mapping of the Mammalian Epitranscriptome,” Current Opinion in Genetics & Development 87 (2024): 102212, 10.1016/j.gde.2024.102212. 38823337

[36] L. Lofeu and E. P. Ahi , “Heterokairic Genes and the Eco‐Evo‐Devo of Timing,” Evolution & Development 28 (2026): 70036, 10.1111/ede.70036. PMC13051254 41937430

[37] P. A. Gameiro , V. Encheva , M. Silva Dos Santos , J. I. Macrae , and J. Ule , “Metabolic Turnover and Dynamics of Modified Ribonucleosides by 13C Labeling,” Journal of Biological Chemistry 297 (2021): 101294, 10.1016/j.jbc.2021.101294. 34634303 PMC8567201

[38] S. R. Millar , J. Q. Huang , K. J. Schreiber , et al., “A New Phase of Networking: The Molecular Composition and Regulatory Dynamics of Mammalian Stress Granules,” Chemical Reviews 123 (2023): 9036–9064, 10.1021/acs.chemrev.2c00608. 36662637 PMC10375481

[39] A. Louloupi , E. Ntini , T. Conrad , and U. A. V. Ørom , “Transient N‐6‐Methyladenosine Transcriptome Sequencing Reveals a Regulatory Role of m6A in Splicing Efficiency,” Cell Reports 23 (2018): 3429–3437, 10.1016/j.celrep.2018.05.077. 29924987

[40] K. E. Pendleton , B. Chen , K. Liu , et al., “The U6 snRNA M 6 A Methyltransferase METTL16 Regulates SAM Synthetase Intron Retention,” Cell 169 (2017): 824–835.e14, 10.1016/j.cell.2017.05.003. 28525753 PMC5502809

[41] K. A. Doxtader , P. Wang , A. M. Scarborough , D. Seo , N. K. Conrad , and Y. Nam , “Structural Basis for Regulation of METTL16, an S‐Adenosylmethionine Homeostasis Factor,” Molecular Cell 71 (2018): 1001–1011.e4, 10.1016/j.molcel.2018.07.025. 30197297 PMC6367934

[42] M. Mendel , K. M. Chen , D. Homolka , et al., “Methylation of Structured RNA by the m6A Writer METTL16 Is Essential for Mouse Embryonic Development,” Molecular Cell 71 (2018): 986–1000.e11, 10.1016/j.molcel.2018.08.004. 30197299 PMC6162343

[43] D. Arango , D. Sturgill , N. Alhusaini , et al., “Acetylation of Cytidine in mRNA Promotes Translation Efficiency,” Cell 175 (2018): 1872–1886. 30449621 10.1016/j.cell.2018.10.030 PMC6295233

[44] G. Jia , Y. Fu , X. Zhao , et al., “N6‐Methyladenosine in Nuclear RNA Is a Major Substrate of the Obesity‐Associated FTO,” Nature Chemical Biology 7 (2011): 885–887, 10.1038/nchembio.687. 22002720 PMC3218240

[45] G. Zheng , J. A. Dahl , Y. Niu , et al., “ALKBH5 is a Mammalian RNA Demethylase That Impacts RNA Metabolism and Mouse Fertility,” Molecular Cell 49 (2013): 18–29. 23177736 10.1016/j.molcel.2012.10.015 PMC3646334

[46] C. Xu , K. Liu , W. Tempel , et al., “Structures of Human ALKBH5 Demethylase Reveal a Unique Binding Mode for Specific Single‐Stranded N6‐Methyladenosine RNA Demethylation,” Journal of Biological Chemistry 289 (2014): 17299–17311, 10.1074/jbc.M114.550350. 24778178 PMC4067165

[47] W. Aik , J. S. Scotti , H. Choi , et al., “Structure of human RNA N 6‐Methyladenine Demethylase ALKBH5 Provides Insights Into Its Mechanisms of Nucleic Acid Recognition and Demethylation,” Nucleic Acids Research 42 (2014): 4741–4754, 10.1093/nar/gku085. 24489119 PMC3985658

[48] R. Su , L. Dong , C. Li , et al., “R‐2HG Exhibits Anti‐Tumor Activity by Targeting FTO/m6A/MYC/CEBPA Signaling,” Cell 172 (2018): 90–105.e23, 10.1016/j.cell.2017.11.031. 29249359 PMC5766423

[49] C. M. Fitzsimmons , M. D. Mandler , J. C. Lunger , et al., “Rewiring of RNA Methylation by the Oncometabolite Fumarate in Renal Cell Carcinoma,” NAR Cancer 6 (2024): zcae004. 38328795 10.1093/narcan/zcae004 PMC10849186

[50] S. Schwartz , D. A. Bernstein , M. R. Mumbach , et al., “Transcriptome‐Wide Mapping Reveals Widespread Dynamic‐Regulated Pseudouridylation of ncRNA and mRNA,” Cell 159 (2014): 148–162, 10.1016/j.cell.2014.08.028. 25219674 PMC4180118

[51] T. M. Carlile , M. F. Rojas‐Duran , B. Zinshteyn , H. Shin , K. M. Bartoli , and W. V. Gilbert , “Pseudouridine Profiling Reveals Regulated mRNA Pseudouridylation in Yeast and Human Cells,” Nature 515 (2014): 143–146. 25192136 10.1038/nature13802 PMC4224642

[52] J. Karijolich , C. Yi , and Y. T. Yu , “Transcriptome‐Wide Dynamics of RNA Pseudouridylation,” Nature Reviews Molecular Cell Biology 16 (2015): 581–585, 10.1038/nrm4040. 26285676 PMC5694666

[53] C. Zhang , L. Chen , D. Peng , et al., “METTL3 and N6‐Methyladenosine Promote Homologous Recombination‐Mediated Repair of DSBs by Modulating DNA‐RNA Hybrid Accumulation,” Molecular Cell 79 (2020): 425–442.e7, 10.1016/j.molcel.2020.06.017. 32615088

[54] J. Hou , Y. Gao , B. Han , S. Yan , S. Wei , and X. Gao , “Nuclear Accumulation of YTHDF1 Regulates mRNA Splicing in the DNA Damage Response,” Science Advances 11 (2025): 7660, 10.1126/sciadv.ado7660. PMC12002136 40238889

[55] H. L. Sun , A. C. Zhu , Y. Gao , et al., “Stabilization of ERK‐Phosphorylated METTL3 by USP5 Increases m6A Methylation,” Molecular Cell 80 (2020): 633–647.e7, 10.1016/j.molcel.2020.10.026. 33217317 PMC7720844

[56] F. Yu , J. Wei , X. Cui , et al., “Post‐Translational Modification of RNA m6A Demethylase ALKBH5 Regulates ROS‐Induced DNA Damage Response,” Nucleic Acids Research 49 (2021): 5779–5797, 10.1093/nar/gkab415. 34048572 PMC8191756

[57] G. Hou , X. Zhao , L. Li , et al., “SUMOylation of YTHDF2 Promotes mRNA Degradation and Cancer Progression by Increasing Its Binding Affinity With m6A‐Modified mRNAs,” Nucleic Acids Research 49 (2021): 2859–2877, 10.1093/nar/gkab065. 33577677 PMC7969013

[58] Y. Li , X. He , X. Lu , et al., “METTL3 Acetylation Impedes Cancer Metastasis via Fine‐Tuning Its Nuclear and Cytosolic Functions,” Nature Communications 13 (2022): 6350, 10.1038/s41467-022-34209-5. PMC9605963 36289222

[59] J. Li , M. Ahmad , L. Sang , et al., “O‐GlcNAcylation Promotes the Cytosolic Localization of the m6A Reader YTHDF1 and Colorectal Cancer Tumorigenesis,” Journal of Biological Chemistry 299 (2023): 104738, 10.1016/j.jbc.2023.104738. 37086786 PMC10208891

[60] Z. Chen , J. Yin , Z. Feng , et al., “O‐GlcNAcylation of METTL3 Drives Hepatocellular Carcinoma Progression by Upregulating MCM10 Expression in an m6A‐IGF2BP3‐Dependent Manner,” Cell Death & Disease 16 (2025): 518, 10.1038/s41419-025-07844-1. 40651967 PMC12255776

[61] D. S. W. Protter and R. Parker , “Principles and Properties of Stress Granules,” Trends in Cell Biology 26 (2016): 668–679, 10.1016/j.tcb.2016.05.004. 27289443 PMC4993645

[62] A. Khong , T. Matheny , T. N. Huynh , V. Babl , and R. Parker , “Limited Effects of m6A Modification on mRNA Partitioning Into Stress Granules,” Nature Communications 13 (2022): 3735, 10.1038/s41467-022-31358-5. PMC9243116 35768440

[63] M. Anders , I. Chelysheva , I. Goebel , et al., “Dynamic M^6^A Methylation Facilitates mRNA Triaging to Stress Granules,” Life Science Alliance 1 (2018); 201800113. 10.26508/lsa.201800113 PMC6238392 30456371

[64] Y. Ge , R. Chen , T. Ling , et al., “Elevated WTAP Promotes Hyperinflammation by Increasing m6A Modification in Inflammatory Disease Models,” Journal of Clinical Investigation 134 (2024): 177932. 10.1172/JCI177932 PMC11245160 39007267

[65] A. Feng , Y. Liang , P. Fu , Y. Dong , S. M. Black , and T. Wang , “Endotoxin‐Induced m6A RNA Methylation Landscape in Lung Endothelial Cells: Role of METTL3 in Regulating Inflammation and Injury During Acute Lung Injury,” Biochimica et Biophysica Acta (BBA)—Molecular Basis of Disease 1871 (2025): 167907, 10.1016/j.bbadis.2025.167907. 40379220 PMC12614311

[66] Z. Zeng , Q. Pan , Y. Sun , et al., “METTL3 Protects METTL14 From STUB1‐Mediated Degradation to Maintain M^6^A Homeostasis,” Embo Reports 24 (2023): EMBR202255762. 10.15252/embr.202255762 PMC9986817 36597993

[67] X. Zhang , Y. Zhang , R. Li , et al., “STUB1‐Mediated Ubiquitination and Degradation of NSUN2 Promotes Hepatocyte Ferroptosis by Decreasing m5C Methylation of Gpx4 mRNA,” Cell Reports 43 (2024): 114885, 10.1016/j.celrep.2024.114885. 39453812

[68] L. Chen , C. Zhang , Y. Ge , et al., “PRKN‐Mediated Ubiquitin‐Proteasome Degradation of METTL3 Promotes Cellular Senescence,” Aging Cell 25 (2026): 70347. 10.1111/acel.70347 PMC12745842 41457744

[69] D. Vukić , A. Cherian , S. Keskitalo , et al., “Distinct Interactomes of ADAR1 Nuclear and Cytoplasmic Protein Isoforms and Their Responses to Interferon Induction,” Nucleic Acids Research 52 (2024): 14184–14204. 39673305 10.1093/nar/gkae1106 PMC11662693

[70] Y. Motorin and M. Helm , “General Principles and Limitations for Detection of RNA Modifications by Sequencing,” Accounts of Chemical Research 57 (2024): 275–288, 10.1021/acs.accounts.3c00529. 38065564 PMC10851944

[71] Y. Liu , Y. Li , Q. Sun , Y. Liu , Q. Sun , and Y. Li , “Advances in Detecting RNA Modifications Using Direct RNA Nanopore Sequencing,” Advanced Genetics 6 (2025): 00041, 10.1002/ggn2.202500041. PMC12747559 41473688

[72] J. Spangenberg , S. Mündnich , A. Busch , et al., “The RMaP Challenge of Predicting RNA Modifications by Nanopore Sequencing,” Communications Chemistry 8 (2025): 115, 10.1038/s42004-025-01507-0. 40221591 PMC11993749

[73] J. Wei , F. Liu , Z. Lu , et al., “Differential m6A, m6Am, and m1A Demethylation Mediated by FTO in the Cell Nucleus and Cytoplasm,” Molecular Cell 71 (2018): 973–985.e5, 10.1016/j.molcel.2018.08.011. 30197295 PMC6151148

[74] L. Kawarada , T. Suzuki , T. Ohira , S. Hirata , K. Miyauchi , and T. Suzuki , “ALKBH1 is an RNA Dioxygenase Responsible for Cytoplasmic and Mitochondrial tRNA Modifications,” Nucleic Acids Research 45 (2017): 7401–7415, 10.1093/nar/gkx354. 28472312 PMC5499545

[75] S. Delaunay , G. Pascual , B. Feng , et al., “Mitochondrial RNA Modifications Shape Metabolic Plasticity in Metastasis,” Nature 607 (2022): 593–603. 35768510 10.1038/s41586-022-04898-5 PMC9300468

[76] Z. Zou , X. Dou , Y. Li , et al., “RNA M^5^C Oxidation by TET2 Regulates Chromatin state and Leukaemogenesis,” Nature 634 (2024): 986–994. 39358506 10.1038/s41586-024-07969-x PMC11499264

[77] R. Wu , C. Sun , X. Chen , et al., “NSUN5/TET2‐Directed Chromatin‐Associated RNA Modification of 5‐Methylcytosine to 5‐Hydroxymethylcytosine Governs Glioma Immune Evasion,” Proceedings of the National Academy of Sciences 121 (2024): 2321611121, 10.1073/pnas.2321611121. PMC10998593 38547058

[78] W. C. Hao , M. J. Dian , Y. Zhou , et al., “Autophagy Induction Promoted by m^6^A Reader YTHDF3 Through Translation Upregulation of FOXO_3_ mRNA,” Nature Communications 13 (2022): 5845. 10.1038/s41467-022-32963-0 PMC9532426 36195598

[79] Y. Chen , X. Liu , L. Li , et al., “Methyltransferase‐Like 3 Aggravates Endoplasmic Reticulum Stress in Preeclampsia by Targeting TMBIM6 in YTHDF2‐Dependent Manner,” Molecular Medicine 29 (2023): 19, 10.1186/s10020-023-00604-x. 36747144 PMC9901113

[80] X. Sun , Q. Tan , Y. Yang , et al., “The Dual Mechanism of m6A Demethylase ALKBH5 in Regulating Energy Metabolism During Exposure to MC‐LR,” Cell Death & Disease 16 (2025): 489, 10.1038/s41419-025-07791-x. 40610445 PMC12229691

[81] N. Zhang , J. Yang , Y. Zhao , et al., “RNA m6A Involves in Regulation of Oxidative Stress and Apoptosis May via NF‐kB Pathway in Cadmium‐Induced Lung Cells,” Cell Death Discovery 11 (2025): 4, 10.1038/s41420-024-02284-w. 39794323 PMC11723944

[82] S. Ke , A. Pandya‐Jones , Y. Saito , et al., “m 6 A mRNA Modifications Are Deposited in Nascent Pre‐mRNA and Are Not Required for Splicing but Do Specify Cytoplasmic Turnover,” Genes & Development 31 (2017): 990–1006, 10.1101/gad.301036.117. 28637692 PMC5495127

[83] M. A. Garcia‐Campos , S. Edelheit , U. Toth , et al., “Deciphering the “m6A Code” via Antibody‐Independent Quantitative Profiling,” Cell 178 (2019): 731–747.e16, 10.1016/j.cell.2019.06.013. 31257032

[84] B. Molinie , J. Wang , K. S. Lim , et al., “m6A‐LAIC‐Seq Reveals the Census and Complexity of the m6A Epitranscriptome,” Nature Methods 13 (2016): 692–698, 10.1038/nmeth.3898. 27376769 PMC5704921

[85] K. D. Meyer , “DART‐Seq: An Antibody‐Free Method for Global M^6^A Detection,” Nature Methods 16 (2019): 1275–1280, 10.1038/s41592-019-0570-0. 31548708 PMC6884681

[86] Y. Y. Xie , Z. D. Zhong , H. X. Chen , et al., “Single‐Molecule Direct RNA Sequencing Reveals the Shaping of Epitranscriptome Across Multiple Species,” Nature Communications 16 (2025): 5119, 10.1038/s41467-025-60447-4. PMC12130510 40456740

[87] M. Tegowski , M. N. Flamand , and K. D. Meyer , “scDART‐Seq Reveals Distinct m6A Signatures and mRNA Methylation Heterogeneity in Single Cells,” Molecular Cell 82 (2022): 868–878.e10, 10.1016/j.molcel.2021.12.038. 35081365 PMC8857065

[88] E. P. Ahi and M. Khorshid , “Potentials of RNA Biosensors in Developmental Biology,” Developmental Biology 526 (2025): 173–188, 10.1016/j.ydbio.2025.07.011. 40721002

[89] L. Zhang , J. Wei , Z. Zou , and C. He , “RNA Modification Systems as Therapeutic Targets,” Nature Reviews Drug Discovery 25 (2025): 59–78. 40962853 10.1038/s41573-025-01280-8

[90] E. Yankova , W. Blackaby , M. Albertella , et al., “Small‐Molecule Inhibition of METTL3 as a Strategy Against Myeloid Leukaemia,” Nature 593 (2021): 597–601. 33902106 10.1038/s41586-021-03536-w PMC7613134

[91] Y. Huang , R. Su , Y. Sheng , et al., “Small‐Molecule Targeting of Oncogenic FTO Demethylase in Acute Myeloid Leukemia,” Cancer Cell 35 (2019): 677–691.e10, 10.1016/j.ccell.2019.03.006. 30991027 PMC6812656

